# Synthesis, DFT calculations, and anti-proliferative evaluation of pyrimidine and selenadiazolopyrimidine derivatives as dual Topoisomerase II and HSP90 inhibitors

**DOI:** 10.1080/14756366.2023.2198163

**Published:** 2023-04-10

**Authors:** Samar El-Kalyoubi, Samiha A. El-Sebaey, A. M. Rashad, Hanan A. AL-Ghulikah, Mostafa M. Ghorab, Sherin M. Elfeky

**Affiliations:** a Department of Pharmaceutical Organic Chemistry, Faculty of Pharmacy, Port Said University, Port Said, Egypt; b Department of Pharmaceutical Organic Chemistry, Faculty of Pharmacy (Girls), Al-Azhar University, Cairo, Egypt; c Accelerator and Ion Sources Department, Nuclear Research Center, Atomic Energy Authority, Cairo, Egypt; d Central Lab for Elemental and Isotopic Analysis, NRC, Atomic Energy Authority, Cairo, Egypt; e Department of Chemistry, College of Science, Princess Nourah Bint Abdulrahman University, Riyadh, Saudi Arabia; f Department of Drug Radiation Research, National Centre for Radiation Research and Technology (NCRRT), Egyptian Atomic Energy Authority (EAEA), Cairo, Egypt; g Department of Pharmaceutical Organic Chemistry, Faculty of Pharmacy, Mansoura University, Mansoura, Egypt

**Keywords:** Pyrimidines, Topoisomerase II, HSP90, Anti-proliferative, *in silico* studies

## Abstract

Novel series of aminopyrimidines bearing a biologically active cyclohexenone **3a–f** and oxo-selaneylidene moiety **4**, besides selenadiazolopyrimidines (**5a–e** and **7**), were synthesised using 5,6-diaminouracils as starting materials. Compound **3a** exhibited strong anti-proliferative activity against three cell lines: HepG-2 (IC_50_ 14.31 ± 0.83 µM), A-549 (IC_50_ 30.74 ± 0.76 µM), and MCF-7 (IC_50_ 27.14 ± 1.91 µM). Also, it was four times more selectively cytotoxic against WI-38 cell lines than doxorubicin. Furthermore, Topoisomerase II (IC_50_ 4.48 ± 0.65 µM) and HSP90 (IC_50_ 1.78 ± 0.11 µM) were both strongly inhibited *in vitro* by **3a**. The cell cycle was halted at the G1-S phase, and total apoptotic cells were 65 times more than control Hep-G2 cells. Besides, it increased caspase-3 gene expression, triggering mitochondrial cell death. Molecular docking study indicated that it could bind to Topoisomerase II and HSP90 binding sites in an inhibitory mode. Its geometric properties were investigated using the density functional theory (DFT). Furthermore, compound **3a** demonstrated *in silico* good oral bioavailability.

## Graphical Abstract

**Figure:**
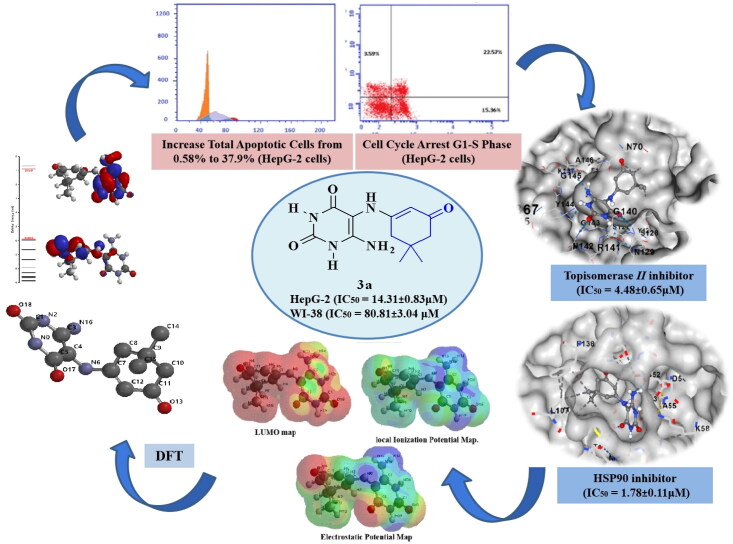

## Introduction

Since cancer cells possess an inherent ability for drug resistance, there is a continuous need for novel potent anti-proliferative agents that target multiple signalling pathways to ensure the effectiveness of cancer therapeutic agents^1^^,^^2^. Pyrimidie-dione (uracil) is a common naturally occurring pyrimidine derivative. Its 5-fluoro derivative was first introduced in the fifties of the last century and is still widely used as an antitumor, especially in colon and breast carcinoma^3^ 5-Flourouracil exhibits its anti-proliferative activity by inhibiting DNA repair and replication^4^. 5-Fluorouracil has been shown to inhibit thymidylate synthase^5^, and it can misincorporateinto DNA in place of uracil, resulting in cytotoxic activity^6^. Different fused uracil derivatives (**I**) showed potent anti-proliferative activity through catalytic inhibition of the Topoisomerase II enzyme and stabilisation of covalent DNA-Topoisomerase II cleavage complexes^7^.

**Figure 1.**
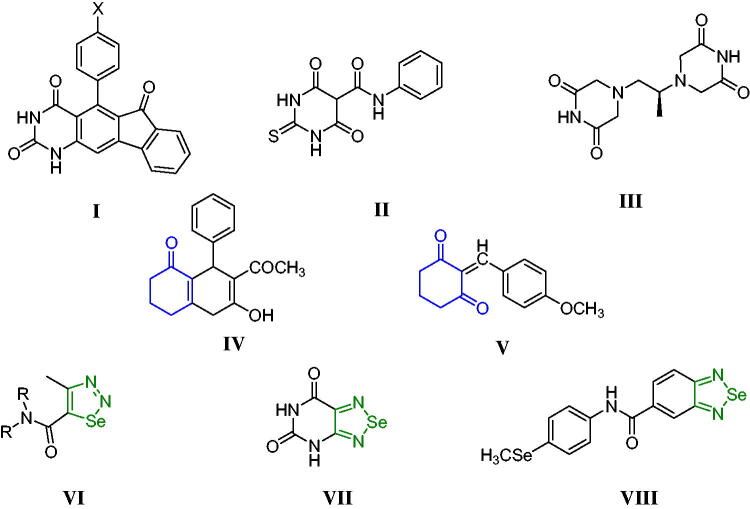
Potent anti-proliferative agents containing pyrimidines, cyclohexenones, and selenadiazoles.

Topoisomerase II inhibitors impair DNA religation and stimulate DNA damage leading to cell cycle arrest. There are two main classes of Topoisomerase II inhibitors. The first class of inhibitors is Topo *II* poisons that target the enzyme through DNA cleavage, including DNA intercalators such as doxorubicin and etoposide. The other class of Topoisomerase II inhibitors is non-competitive inhibitors of ATP, such as merbarone (**II**) and dexrazoxane (**III**)^8^^,^^9^ (Figure 1).

Heat shock protein, HSP90, is an ATP-dependent chaperone that is essential for various processes, such as the folding and degradation of proteins involved in cell proliferation, angiogenesis, and metastasis^10^^,^^11^. It has been reported that HSP90 inhibition results in the suppression of cancer cell growth and proliferation^12^. Unlike other drug targets, HSP90 is expressed 10-fold more in tumour cells than in normal cells, making it a selective target for developing novel anti-proliferative agents^13^.

Since Topoisomerase II inhibitors act through induction of DNA damage with subsequent apoptotic effect^14^, DNA repair system proteins would counter cell death and present a possible resistance mechanism to Topoisomerase II inhibitors^15^. HSP90 inhibitors can suppress DNA repair, so compounds with dual activity as Topoisomerase II and HSP90 inhibitors would have improved potency and limited resistance as anti-proliferative agents^16^^,^^17^. It is worth noting that Topoisomerase II and HSP90 share a nucleotide binding fold as ATP-dependent proteins^18^. Furthermore, it has been reported that inhibiting Hsp90 makes cells susceptible to a Topoisomerase II inhibitor, resulting in cell death *via* apoptosis^19^.

Cyclohexanone, particularly its dimethyl derivative, is an important class and precursor for various anti-proliferative agents, with (**IV**) and (**V**) demonstrating potent antitumor activity against a variety of tumours and cell lines^20^ (Figure 1). The Key trace element selenium can be incorporated into polypeptide chains to form seleno-proteins, which have important roles and functions such as anti-inflammatory, antiviral, chemopreventive, and immune system-improving properties as well as cancer prevention thanks to their strong antioxidant properties^21–24^. In humans, selenium is incorporated into the selenoprotein as the amino acid selenocysteine. One of the most important selenoproteins is Glutathione peroxidase (GPX-1), a crucial detoxification and antioxidant enzyme^25^^,^^26^. Selenium-containing organic compounds have been reported to have anti-proliferative properties through the induction of apoptosis. Also, selenium compounds have been shown to influence DNA repair, angiogenesis, and metastasis. They induce cell arrest, necrosis, autophagy, and necroptosis, making them promising candidates for tumour cell death induction^27^. Selenadiazoles, as selenium-containing ring systems, have been reported to be potent anti-proliferative agents; 4-methyl-1,2,3-selenadiazole-5-carboxylic acid amides (**VI**) demonstrated both *in vitro* and *in vivo* against anti-proliferative activities^28^. Furthermore, 1,2,5-selenadiazolo-[3,4-*d*]pyrimidine-5,7-(4*H*,6*H*)-dione (**VII**) was reported to have cytotoxic activity against melanoma, hepatoma, and breast cancer cells^29^. Selenadiazole compound (**VIII**) was reported to have potent anti-proliferative activity with selective cytotoxicity and radical scavenging properties^30^ (Figure 1).

**Figure 2.**
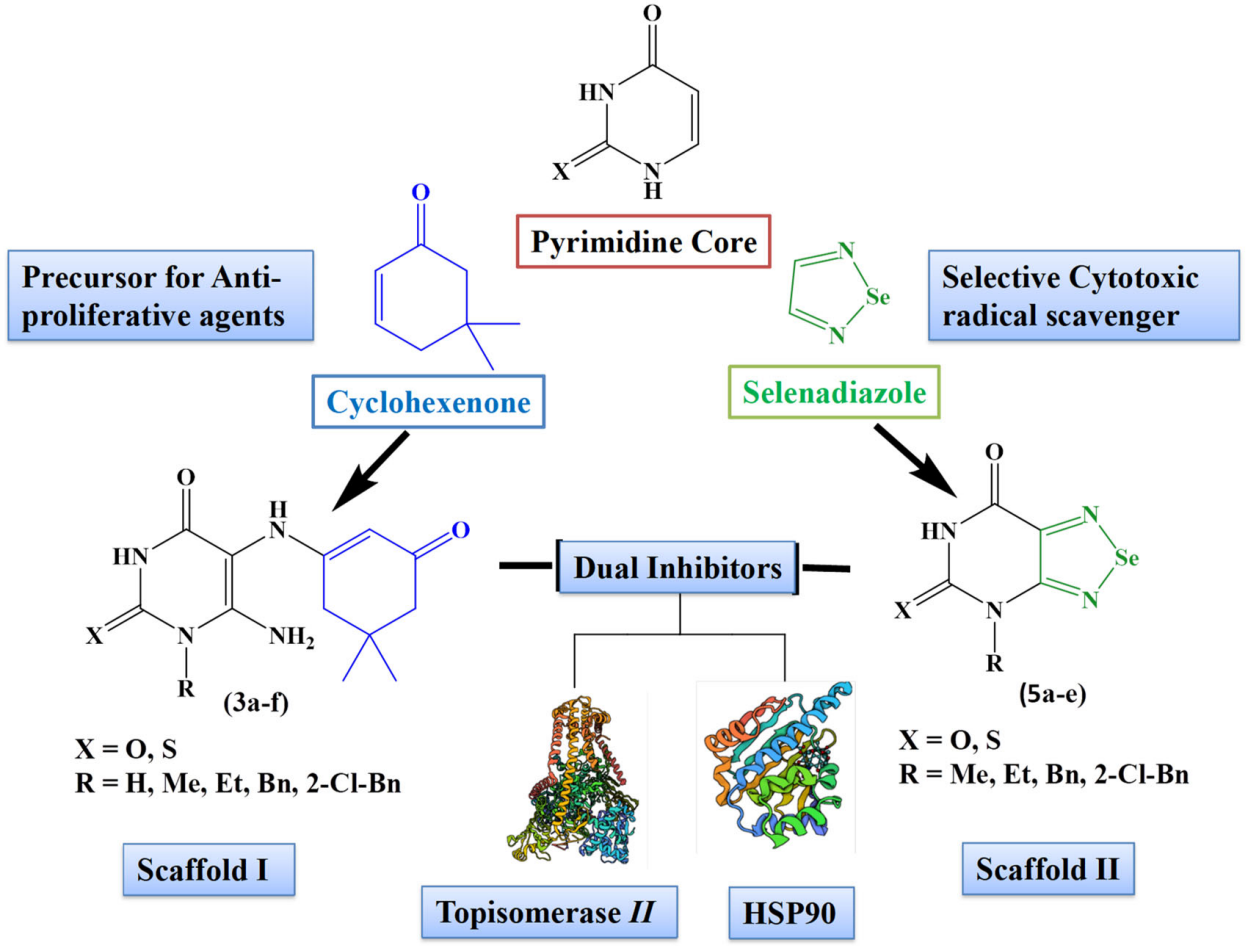
Design of scaffolds I and II as dual inhibitors of Topoisomerase *II* and HSP90.

In the present investigation and in continuation of our ongoing efforts to discover potent anti-proliferative agents^31^^,^^32^, uracil/thiouracil was used as the core for designing two scaffolds as dual Topoisomerase II and HSP90 inhibitors, as shown in Figure 2. Scaffold I is a cyclohexenone-pyrimidine hybrid in which a 5,5-dimethyl cyclohexanone moiety was introduced at the pyrimidine moiety through an NH linker, along with the introduction of the amino group at position-6 of pyrimidine ring to act as hydrogen bond donner and various alkyl/aryl-alkyl groups at *N.* While, scaffold II (selenadiazole-pyrimidine hybrid) is made up of a selenadiazole ring system fused to a pyrimidine core with different alkyl/aryl-alkyl groups at *N*1 and *N*3.

## Materials and methods

### Chemistry

Stuart melting point apparatus (SMP 30) was used to measure melting points (°C) and are uncorrected. The reactions were monitored using pre-coated (0.25 mm) silica gel plates (Merck 60 F_254_, Germany), and the spots were visualised using a UV lamp (254 nm). Chloroform: methanol (9:1) and ethyl acetate: toluene (1:1) was used as elution systems. NMR spectra were recorded in (DMSO) at ^1^HNMR (400 MHz) and ^13^CNMR (100 MHz) using TMS as an internal standard on a Bruker NMR spectrometer (*δ* ppm), Zagazig university. Mass spectra were performed on the direct inlet part of the mass analyser in a Thermo Scientific GCMS model ISQ in the Regional Centre for Mycology and Biotechnology (RCMB), Al-Azhar University, Egypt. Energy-dispersive X-ray spectroscopy (EDX analysis) was carried out by scanning electron microscope (SEM) connected to a LaB6 electron gun (Philips-EDAX/DX4) energy-dispersive spectroscope (EDX), National Research Centre, Dokki, Giza, Egypt. Aldrich Chemicals Co., USA, as well as commercial sources, provided all of the chemicals and reagents used.

#### General method for preparation of 6-amino-5-((5,5-dimethyl-3-oxocyclohex-1-en-1-yl)amino)pyrimidines (3a–f)

An equimolar amount of 5,6-diaminouracils (**1a–f)** (1 mmol) and dimedone (**2**) (1 mmol) in dimethylformamide (DMF, 1 ml) were heated under fusion for 12–15 min. The reaction was allowed to cool before adding methanol, and the formed precipitate was collected by filtration, washed with methanol, and recrystallized from DMF-ethanol, affording the desired compounds (**3a–f**) in good yields.

##### 6-Amino-5-((5,5-dimethyl-3-oxocyclohex-1-en-1-yl)amino)pyrimidine-2,4(1*H*,3*H*)-dione (3a)

Light yellow solid, yield: 65%; m.p. >300 °C; ^1^H NMR (400 MHz, DMSO-*d*6) *δ* 10.40 (s, 1H, NH, D_2_O exchangeable), 10.29 (s, 1H, NH, D_2_O exchangeable), 7.33 (s, 1H, NH, D_2_O exchangeable), 6.04 (s, 2H, NH_2_, D_2_O exchangeable), 4.58 (s, 1H, cyclohexenone), 2.33 (br s, 1H, cyclohexenone), 2.26 (br s, 1H, cyclohexenone), 1.95 (s, 2H, CH_2,_ cyclohexenone), 0.99 (s, 6H, 2CH_3_). ^13^C NMR (100 MHz, DMSO-*d*6) *δ* 194.7, 161.9, 160.0, 155.3, 149.9, 95.3, 85.7, 50.6, 41.6, 32.5, 28.2, 27.8 ppm. MS: *m/z* (%) = M^+^, 264 (25), 250 (25), 248 (35), 229 (45), 227 (33), 209 (39), 206 (30), 193 (36), 191 (72), 177 (68), 139 (47), 114 (60), 113 (42), 112 (51), 104 (70), 103 (60), 102 (70), 100 (84), 99 (100), 90 (53), 77 (82), 73 (50); Anal. calc. for C_12_H_16_N_4_O_3_ (264.29): C, 54.54; H, 6.10; N, 21.20; Found: C, 54.68; H, 6.; N, 21.47.

##### 6-Amino-1-(2-chlorobenzyl)-5-((5,5-dimethyl-3-oxocyclohex-1-en-1-yl)amino)pyrimidine-2,4(1*H*,3*H*)-dione (3b)

Light yellow solid, yield: 60%; m.p. = 294–296 °C; ^1^H NMR (400 MHz, DMSO-*d*6) *δ* 10.80 (s, 1H, NH), 7.41 (s, 1H, NH), 7.35 (t, *J* = 7.3 Hz, 1H, ArH), 7.27 (t, *J* = 7.3 Hz, 1H, ArH), 7.20 (d, *J* = 7.3 Hz, 2H, ArH), 6.63 (s, 2H, NH_2_), 5.15 (d, *J* = 15.5 Hz, 1H, CH_2_, benzyl), 5.09 (d, *J* = 15.5 Hz, 1H, CH_2_, benzyl) 4.62 (s, 1H, cyclohexenone), 2.43 (d, *J* = 14.0 Hz, 1H, cyclohexenone), 2.21 (d, *J* = 14.0 Hz, 1H, cyclohexenone), 1.96 (s, 2H, CH_2_, cyclohexenone), 0.99 (s, 6H, 2CH_3_).^13^C NMR (100 MHz, DMSO-*d*6) *δ* 195.1, 164.8, 159.9, 153.1, 150.8, 136.9, 128.9 (2), 127.7, 126.6 (2), 95.9, 88.6, 51.1, 44.8, 42.0, 32.8, 29.5, 28.0 ppm. MS: *m/z* (%) = M^+^+2, 391 (5), M^+^, 389 (21), 387 (48), 386 (89), 306 (62), 251 (28), 247 (100), 245 (21), 243 (40), 240 (17), 148 (54), 135 (98); Anal. calc. for C_19_H_21_ClN_4_O_3_ (388.85): C, 58.69; H, 5.44; N, 14.41; Found: C, 58.90; H, 5.67; N, 14.70.

##### 6-Amino-5-((5,5-dimethyl-3-oxocyclohex-1-en-1-yl)amino)-1-methyl-2-thioxo-2,3-dihydropyrimidin-4(1*H*)-one (3c)

Light yellow solid, yield: 61%; m.p. >300 °C; ^1^H NMR (400 MHz, DMSO-*d*6) *δ* 12.11 (s, 1H, NH), 7.53 (s, 1H, NH), 6.85 (s, 2H, NH_2_), 4.59 (s, 1H, cyclohexenone), 3.74 (s, 3H, N–CH_3_), 2.43 (br s, 1H, cyclohexenone**),** 2.24 (br s, 1H, cyclohexenone**),** 1.96 (s, 2H, CH_2_, cyclohexenone), 1.00 (s, 6H, 2CH_3_). ^13^C NMR (100 MHz, DMSO-*d*6) *δ* 194.9, 174.9, 163.6, 157.0, 153.0, 95.7, 92.9, 50.4, 41.5, 36.1, 32.5, 28.9, 27.7 ppm. MS: *m/z* (%) = M^+^, 294 (12), 284 (73), 254 (51), 207 (100), 88 (64), 86 (66), 80 (61); Anal. calc. for C_13_H_18_N_4_O_2_S (294.37): C, 53.04; H, 6.16; N, 19.03; Found: C, 53.26; H, 6.40; N, 19.29.

##### 6-Amino-5-((5,5-dimethyl-3-oxocyclohex-1-en-1-yl)amino)-1-ethylpyrimidine-2,4(1*H*,3*H*)-dione (3d)

Olive solid, yield: 94%; m.p. >300 °C; ^1^H NMR (400 MHz, DMSO-*d*6) *δ* 10.61 (s, 1H, NH, D_2_O exchangeable), 7.37 (s, 1H, NH, D_2_O exchangeable), 6.64 (s, 2H, NH_2,_ D_2_O exchangeable), 4.57 (s, 1H, cyclohexenone), 3.84 (q, *J* = 7.0 Hz, 2H, N–CH_2_ Et), 2.45 (d, *J* = 15.3 Hz, 1H, cyclohexenone), 2.21 (d, *J* = 15.3 Hz, 1H, cyclohexenone), 1.96 (s, 2H, CH_2_, cyclohexenone), 1.11 (t, *J* = 7.0 Hz, 3H, CH_3_, Et), 0.99 (s, 6H, 2CH_3_). ^13^C NMR (100 MHz, DMSO-*d*6) *δ* 194.6, 164.3, 158.8, 152.0, 149.8, 95.1, 87.8, 50.6, 41.5, 36.8, 32.4, 29.0, 27.6, 13.2 ppm. MS: *m/z* (%) = M^+^, 292 (28), 288 (67), 257 (56), 248 (45), 238 (47), 233 (64), 215 (52), 166 (77), 111 (49), 103 (47), 77 (100), 76 (62); Anal. calc. for C_14_H_20_N_4_O_3_ (292.34): C, 57.52; H, 6.90; N, 19.17; Found: C, 57.71; H, 7.12; N, 19.45.

##### 6-Amino-1-benzyl-5-((5,5-dimethyl-3-oxocyclohex-1-en-1-yl)amino)pyrimidine-2,4(1*H*,3*H*)-dione (3e)

Yellow solid, yield: 70%; m.p. = 292–294 °C; ^1^H NMR (400 MHz, DMSO-*d*6) *δ* 10.80 (s, 1H, NH), 7.40 (s, 1H, NH), 7.35 (t, *J* = 7.4 Hz, 2H, ArH), 7.27 (t, *J* = 7.4 Hz, 1H, ArH), 7.20 (d, *J* = 7.4 Hz, 2H, ArH), 6.63 (s, 2H, NH_2_), 5.15 (d, *J* = 12.6 Hz, 1H, CH_2_, benzyl), 5.09 (d, *J* = 12.6 Hz, 1H, CH_2_, benzyl), 4.61 (s, 1H, cyclohexenone), 2.43 (d, *J* = 15.4 Hz, 1H, cyclohexenone), 2.21 (d, *J* = 15.4 Hz, 1H, cyclohexenone), 1.95 (s, 2H, CH_2_, cyclohexenone), 0.99 (s, 6H, 2CH_3_). ^13^C NMR (100 MHz, DMSO-*d*6) *δ* 194.8, 164.5, 159.5, 152.7, 150.4, 136.5, 128.6 (2), 127.3, 126.2 (2), 95.4, 88.2, 50.6, 44.4, 41.6, 32.4, 29.1, 27.6 ppm. MS: *m/z* (%) = M^+^, 354 (43), 349 (58), 335 (45), 331 (100), 327 (97), 314 (75), 265 (79), 259 (61), 251 (86), 191 (53); Anal. calc. for C_19_H_22_N_4_O_3_ (354.41): C, 64.39; H, 6.26; N, 15.81; Found: C, 64.18; H, 6.43; N, 16.05.

##### 6-Amino-5-((5,5-dimethyl-3-oxocyclohex-1-en-1-yl)amino)-1-methylpyrimidine-2,4(1*H*,3*H*)-dione (3f)

Cinnamon solid, yield: 64%; m.p. >300 °C; ^1^H NMR (400 MHz, DMSO-*d*6) *δ* 10.62 (s, 1H, NH), 7.38 (s, 1H, NH), 6.61 (s, 2H, NH_2_), 4.60 (s, 1H, cyclohexenone), 3.24 (s, 3H, N–CH_3_), 2.45 (d, *J* = 14.5 Hz, 1H, cyclohexenone), 2.22 (d, *J* = 14.5 Hz, 1H, cyclohexenone), 1.95 (s, 2H, CH_2_, cyclohexenone), 0.99 (s, 6H, 2CH_3_), ^13^C NMR (100 MHz, DMSO-*d*6) *δ* 194.6, 164.4, 159.4, 153.3, 150.1, 95.4, 87.9, 50.6, 41.5, 32.4, 29.2, 29.0, 27.6 ppm. MS: *m/z* (%) = M^+^, 278 (28), 255 (63), 215 (60), 194 (74), 162 (100), 142 (88), 78 (82); Anal. calc. for C_13_H_18_N_4_O_3_ (278.31): C, 56.10; H, 6.52; N, 20.13; Found: C, 56.43; H, 6.70; N, 20.29.

##### 6-Amino-1–(2-chlorobenzyl)-5-((oxo-λ^4^-selaneylidene)amino)pyrimidine-2,4(1*H*,3*H*)-dione (4)

Equimolar amounts of 5,6-diaminouracil **1b** (0.3 gm, 0.84 mmol) and selenium dioxide (0.94 gm, 0.84 mmol) in DMF (1 ml) were heated under fusion for 3 min. Methanol was added after cooling, and the forming precipitate was filtered, washed with methanol, dried in the oven, and recrystallized from DMF-ethanol to yield the desired compound **4**.

Light yellow solid, yield: 56%; m.p. >300 °C; ^1^H NMR (400 MHz, DMSO-*d*6) *δ* 10.13 (s, 1H, NH), 7.73 (s, 2H, NH_2_), 7.52 − 7.49 (m, 1H, ArH), 7.34 − 7.31 (m, 2H, ArH), 6.87 − 6.85 (m, 1H, ArH), 5.10 (s, 2H, CH_2_, benzyl), ^13^C NMR (100 MHz, DMSO-*d*6) *δ* 163.5, 158.6, 150.2, 133.4, 131.7, 129.5, 128.7, 127.5, 125.4, 78.4, 44.4 ppm. MS: *m/z* (%) = M^+^+2, 361 (29), M^+^, 359 (33), 355 (29), 351 (23), 349 (45), 331 (18), 329 (47), 316 (72), 308 (52), 300 (37), 298 (85), 230 (82), 210 (46), 192 (46), 110 (43), 85 (53), 71 (100); Anal. calc. for C_11_H_9_ClN_4_O_3_Se (359.95): C, 36.74. 5; H, 2.52; N, 15.58; Found: C, 36.60; H, 2.31; N, 15.75.

#### General preparation of 1,2,5- selenadiazolo[3,4-d]pyrimidine-5,7(4H,6H)-dione derivatives 5a–e

5,6-Diaminouracils **1a** & **1c-f** (1.2 mmol) and selenium dioxide (1.2 mmol) in DMF (1 ml) were heated under fusion for 13–15 min. After cooling, methanol was added, and the formed precipitate was filtered, washed with methanol, dried in the oven, and recrystallized from DMF-ethanol, affording the desired compound **5a-e** in good yields.

##### [1, 2, 5]selenadiazolo[3,4-*d*]pyrimidine-5,7(4*H*,6*H*)-dione (5a)

Buff solid, yield: 60%; m.p. >300 °C; ^1^H NMR (400 MHz, DMSO-*d*6) *δ* 11.93 (s, 1H, NH), 11.48 (s, 1H, NH), ^13^C NMR (100 MHz, DMSO-*d*6) *δ* 156.4, 155.7, 150.5, 144.7 ppm. MS: *m/z* (%) = M^+^, 217 (17), 210 (55), 209 (41), 195 (45), 180 (53), 149 (47), 137 (65), 122 (59), 106 (54), 104 (100), 98 (58), 59 (60); Anal. calc. for C_4_H_2_N_4_O_2_Se (217.06): C, 22.13; H, 0.93; N, 25.81; Found: C, 22.40; H, 1.21; N, 26.09.

##### 4-Methyl-5-thioxo-5,6-dihydro-[1,2,5]selenadiazolo[3,4-*d*]pyrimidin-7(4*H*)-one (5b)

Yellow solid, yield: 79%; m.p. >300 °C; ^1^H NMR (400 MHz, DMSO-*d*6) *δ* 12.66 (s, 1H, NH), 3.87 (s, 3H, N–CH_3_), ^13^C NMR (100 MHz, DMSO-*d*6) *δ* 176.3, 156.6, 153.9, 145.2, 36.5 ppm. MS: *m/z* (%) = M^+^, 247 (12), 197 (87), 173 (98), 147 (52), 95 (100); Anal. calc. for C_5_H_4_N_4_OSSe (247.15): C, 24.30; H, 1.63; N, 22.67; Found: C, 24.56; H, 1.78; N, 22.75.

##### 4-Ethyl-[1, 2, 5]selenadiazolo[3,4-*d*]pyrimidine-5,7(4*H*,6*H*)-dione (5c)

Buff solid, yield: 68%; m.p. >300 °C; ^1^H NMR (400 MHz, DMSO-*d*6) *δ* 11.65 (s, 1H, NH), 4.03 (q, *J* = 7.1 Hz, 2H, N–CH_2_, Et), 1.22 (t, *J* = 7.1 Hz, 3H, CH_3_, Et), ^13^C NMR (100 MHz, DMSO-*d*6) *δ* 155.9, 155.8, 150.1, 144.7, 38.5, 12.5 ppm. MS: *m/z* (%) = M^+^, 245 (16), 236 (46), 224 (46), 223 (46), 208 (39), 179 (43), 177 (50), 174 (66), 155 (69), 154 (60), 147 (100), 143 (57), 142 (74), 134 (98), 109 (50), 97 (47); Anal. calc. for C_6_H_6_N_4_O_2_Se (245.11): C, 29.40; H, 2.47; N, 22.86; Found: C, 29.67; H, 2.68; N, 23.14.

##### 4-Benzyl-[1, 2, 5]selenadiazolo[3,4-*d*]pyrimidine-5,7(4*H*,6*H*)-dione (5d)

Cimon solid, yield: 60%; m.p. = 260–261 °C; ^1^H NMR (400 MHz, DMSO-*d*6) *δ* 11.84 (s, 1H, NH), 7.37 (d, *J* = 7.0 Hz, 2H, ArH), 7.33–7.27 (m, 2H, ArH), 7.26–7.21 (m, 1H, ArH), 5.19 (s, 2H, CH_2_, benzyl), ^13^C NMR (100 MHz, DMSO-*d*6) *δ* 156.1, 155.8, 150.6, 144.6, 136.5, 128.4 (2), 127.2 (3), 46.6 ppm. MS: *m/z* (%) = M^+^, 307 (28), 297 (58), 274 (80), 189 (98), 88 (100), 52 (62); Anal. calc. for C_11_H_8_N_4_O_2_Se (307.18): C, 43.01; H, 2.63; N, 18.24; Found: C, 43.20; H, 2.75; N, 18.40.

##### 4-Methyl-[1, 2, 5]selenadiazolo[3,4-*d*]pyrimidine-5,7(4*H*,6*H*)-dione (5e)

Light brown solid, yield: 69%; m.p. >300 °C; ^1^H NMR (400 MHz, DMSO-*d*6) *δ* 11.53 (s, 1H, NH), 3.42 (s, 3H, N–CH_3_), ^13^C NMR (100 MHz, DMSO-*d*6) *δ* 156.7, 155.7, 150.5, 144.5, 30.1 ppm. MS: *m/z* (%) = M^+^, 231 (38), 209 (55), 164 (46), 147 (69), 136 (78), 135 (69), 131 (97), 129 (95), 122 (77), 116 (68), 109 (65), 95 (68), 60 (52), 48 (100); Anal. calc. for C_5_H_4_N_4_O_2_Se (231.08): C, 25.99; H, 1.74; N, 24.25; Found: C, 26.17; H, 2.02; N, 24.47.

##### 4,6-Dimethyl-[1, 2, 5]selenadiazolo[3,4-*d*]pyrimidine-5,7(4*H*,6*H*)-dione (7)

To a suspension solution of 5,6-diamino-1,3-dimethyluracil hydrochloride (**6**) (0.3 gm, 1.45 mmol) in ethanol, triethylamine (TEA) was added and stirred at room temperature for 15 min before adding SeO_2_ (0.16 gm, 1.45 mmol) with continuous stirring for 1 h. The formed precipitate was collected by filtration, washed with methanol, dried in an oven, and recrystallized from DMF-ethanol.

Paige solid, yield: 76%; m.*p* = 278–280 °C; ^1^H NMR (400 MHz, DMSO-*d*6) *δ* 3.51 (s, 3H, N–CH_3_), 3.27 (s, 3H, N–CH_3_), ^13^C NMR (100 MHz, DMSO-*d*6) *δ* 156.6, 155.9, 151.5, 144.0, 31.8, 29.3 ppm. MS: *m/z* (%) = M^+^, 245 (12), 237 (49), 209 (100), 208 (87), 205 (93), 145 (65), 128 (55), 126 (51), 124 (71), 90 (73), 54 (70); Anal. calc. for C_6_H_6_N_4_O_2_Se (245.11): C, 29.40; H, 2.47; N, 22.86; Found: C, 29.68; H, 2.61; N, 22.97.

### Biological evaluation

#### *In vitro* cytotoxicity assay

The viability assay (MTT method)^33^ was used to evaluate the cytotoxic activity of the synthesised compounds against three mammalian tumour cell lines obtained from the VACSERA Tissue Culture Unit, namely MCF-7 cells (human breast cancer cell line), HepG-2 cells (human Hepatocellular carcinoma), and A-549 cells (lung carcinoma), as well as the normal WI-38 human cell line for both compounds **3a** and **5d**. Chemicals used, such as crystal violet, dimethyl sulfoxide (DMSO), and trypan blue dye (St. Louis, Mo., USA), were provided by Sigma. Lonza provided foetal bovine serum, HEPES buffer solution, DMEM, RPMI-1640, gentamycin, L-glutamine, and 0.25% Trypsin-EDTA. 1% Crystal violet stain: it is composed of 0.5% (w/v) crystal violet and 50% methanol, which is then diluted to volume with ddH_2_O and filtered through a Whatman No.1 filter paper. Dulbecco’s modified Eagle’s medium (DMEM) supplemented with 10% heat-inactivated foetal bovine serum, 1% L-glutamine, HEPES buffer, and 50 μg/mL gentamycin was used to grow the cells. All cells were cultured twice a week and kept at 37 °C in a humidified atmosphere with 5% CO_2_. In 100 μL of the growth medium, the cells were seeded in a 96-well plate at a cell concentration of 1 × 10^4^ cells per well. After 24 h of seeding, a fresh medium containing different concentrations of the test sample was added. A multichannel pipette was used to add serial two-fold dilutions of the tested chemical compound to confluent cell monolayers dispensed into 96-well, flat-bottomed microtiter plates (Falcon, NJ, USA). The microtiter plates were incubated for 24 h at 37 °C in a humidified incubator with 5% CO_2_. For each concentration of the test sample, three wells were used. In the absence of a test sample, control cells were incubated with or without DMSO. The small amount of DMSO present in the wells (maximum 0.1%) had no effect on the experiment. A colorimetric method was used to determine viable cell yield after 24 h of incubation at 37 °C. Following the completion of the incubation period, the media were aspirated, and the crystal violet solution (1%) was added to each well for at least 30 min. The stain was removed, and the plates were rinsed with tap water to remove any remaining stains. Glacial acetic acid (30%) was thoroughly mixed into all wells, and the absorbance of the plates was measured. Background absorbance detected in wells without added stain was corrected for in all results. In the absence of the tested compounds, treated samples were compared to cell controls. All experiments were conducted in triplicate. Each tested compound’s cytotoxic effect on cells was calculated. To determine the number of viable cells, the optical density was measured using a microplate reader (Sunrise, TECAN, Inc, USA), and the percentage of viability was calculated as [(ODt/ODc)] × 100%, where ODt is the mean optical density of wells treated with the tested sample and ODc is the mean optical density of untreated cells. The survival curve of each tumour cell line after treatment with the specified compound is obtained by plotting the relationship between surviving cells and drug concentration. The 50% inhibitory concentration (IC_50_), or the concentration required to cause toxic effects in 50% of intact cells, was calculated using GraphPad Prism software from graphic plots of the dose-response curve for each concentration (San Diego, CA. USA).

#### *In vitro* enzyme inhibition assay

*In vitro* enzyme inhibitory assay of compounds **3a** and **5d** was performed against Topoisomerase-*II* enzyme using Topoisomerase II Assay Kit (plasmid-based)^34^ and HSP90 enzyme using HSP90 (*C*-Terminal) Inhibitor Screening Assay Kit^35^. The procedure of the used kits was done according to the manufacturer’s instructions. Doxorubicin (Topoisomerase II inhibitor) and geldanamycin (HSP90 inhibitor) were used as positive controls.

#### Flow cytometric analysis of cell-cycle distribution

Cell-cycle analysis was performed by DNA staining with propidium iodide (PI) following the manufacturer’s protocol (Abcam, USA)^36^. HepG-2 cells were cultured following the procedure till exponential growth was reached. After removing the culture media, the cells were rinsed with phosphate buffer. Cells were then treated with **3a** and suspended in 66% ethanol on the ice at 4 °C. Then, PI was added together with the RNase staining solution. Incubation was allowed for 20–30 min at 37 °C in the dark. For the preparation of flow cytometry, fixed cells were placed on ice. Podium iodide fluorescence was collected using 488 nm laser illumination.

#### Cellular apoptosis analysis

Annexin V is a protein that has a high affinity to bind to membrane phosphatidylserine (PS) translocated from the inner cellular plasma membrane to the cell surface after apoptosis initiation following the manufacturer protocol (Biovision, USA)^37^. HepG-2 cells in exponential growth were treated with compound **3a** and incubated for 24 h. Then, 1–5 × 10^5^ were collected *via* centrifugation and suspended in 500 µL of 1X Binding Buffer, 5 µL of Annexin V-FITC, and 5 µL of propidium iodide were added and allowed to incubate in the dark for 5 min, then Annexin V-FITC binding was quantified by flow cytometry using emission signal detector at 530 nm.

#### Estimation of Topoisomerase II, HSP90, and caspase-3 gene expression

The level of Topoisomerase II, HSP90, and caspase-3 gene expression were assessed using Bio-Rad RT-PCR Kit following the manufacturer’s instructions^38^. HepG-2 cells were exponentially grown and then treated with **3a** after treatment with DNA polymerase and fluorescein. The expression of Topoisomerase II, HSP90, and caspase-3 genes was measured fluorometrically.

### Molecular docking simulation and molecular modelling

#### Molecular docking simulation

Docking was carried out as described in the literature^39^. Topoisomerase II (PDB ID: 1QZR)^40^ and HSP90 (PDB ID: 1YET)^41^ crystallographic structures were prepared for molecular docking by removing the ligand, adding hydrogens, and minimising energy using MOE software 2009. The energy-efficient structure was also used as a docking receptor. The MOE site finder algorithm was used to identify the Topoisomerase II and HSP90 catalytic sites. ChemBioOffice was used to generate the two-dimensional structures of the synthesised compounds, which were then constructed from fragment libraries in MOE 2009, and energy was minimised in MOE using the MMFF94x force field. To identify and assess the interaction between ligands and the binding sites of Topoisomerase II and HSP90, docking was performed with specific parameters (Rescoring function 1 and Rescoring function 2: London dG, Placement: Triangle matcher, retain: 2, and Refinement: Force field). Based on native ligand S-scores and root-mean-square deviation (RMSD) values, the most efficient hits were chosen. The retrieved compounds had a higher S-value and a lower RMSD.

#### Molecular modelling

**3a** was geometry optimised using SPARTAN '18 (Win/64b) Release 1.4.2, (Spartan 18 Wavefunction Inc. Irvine CA). In these calculations, density functional theory (DFT) with the B3LYP method^42^ was used, with a basis set (6–31 G*)^43^. Bond lengths and angles, as well as the Mulliken atomic charges, were calculated. The highest occupied molecular orbital (HOMO) and the lowest non-occupied molecular orbital (LUMO) were calculated, as well as the related parameters like the ionisation potential (I), electron affinity (A), the electronegativity (χ), global chemical hardness (η), electronic chemical potential (μ), and the molecular electrostatic potential (MEP)^44^.

## Results and discussion

### Chemistry

**Scheme 1.**
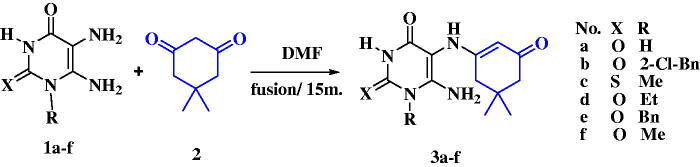
Synthesis of compounds **3a–f.**

**Scheme 2.**
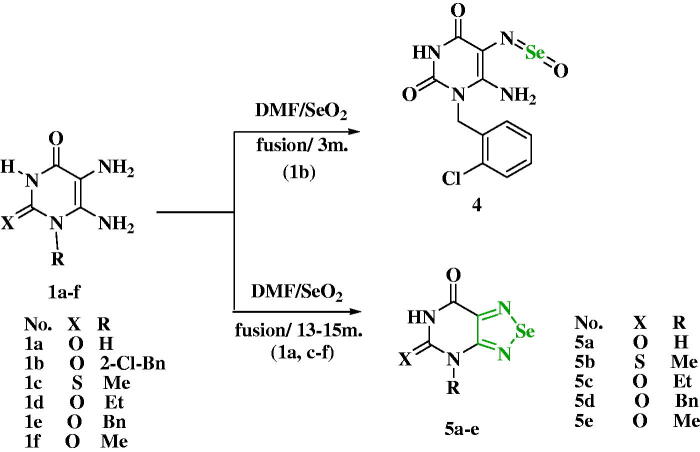
Synthesis of compounds **4** and **5a–e.**

**Scheme 3.**
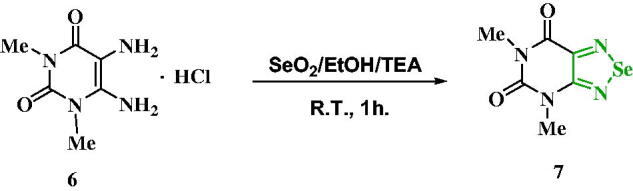
Synthesis of compound **7.**

The sequence of chemical synthesis reactions clarified in Schemes 1, 2, and 3 utilised the starting 5,6-diaminouracils **1a–f** and **6**^45–47^ for the synthesis of the hybrids. A facile one-pot condensation of two-component takes place in DMF without catalyst between 5,6-diaminouracil derivatives **1a–f** and dimedone **2,** affording compounds **3a–f** that were isolated in good yields (Scheme 1).

TLC revealed a single product in all cases, which could be fully characterised by analytical and spectroscopic data. Regarding the heterocyclic unit, for example, the ^1^H NMR spectrum of compound **3d** exhibits relatively three sharp singlet signals at δ 10.61, 7.37, and 6.64 ppm, which correspond to the NH-3, NH-5, and NH_2_-6 groups, respectively. The three former ones disappeared using D_2_O. Also, a singlet signal at δ4.57 ppm characterised for α-CH of α,β- unsaturated ketone of oxocyclohexenyl ring appears. The two protons of CH_2_ in the oxocyclohexenyl ring show a diastereotopic proton with different chemical shift values at δ 2.45 and 2.21 ppm. The mechanistic pathway for the above reaction takes place by the nucleophilic attack of NH_2_-5 of uracils to the electrophilic carbon centre of the carbonyl group of dimedone, followed by 1,2-proton migration to yield the non-isolable carbinolamine intermediate. The latter intermediate is protonated and converted into a better-leaving group, which is then eliminated as water, producing an iminium ion and finally resulting in α-β unsaturated ketone **3a–f** through an Elimination Unimolecular conjugate Base (E1cB) mechanism. Furthermore, the reactivity of strategic starting materials **1a–f** towards SeO_2_ was investigated. An optimised microwave-assisted solid-state synthesis method was used for the synthesis of 1,2,5-selenadiazolo-[3,4-*d*]pyrimidine-5,7-(4*H*,6*H*)-dione from 5,6-diaminouracil by Chen T. et al.^29^. Additionally, selenadiazolopyrimidines^48^^,^^49^ were also prepared *via* the reaction of 5,6-diaminouracil with selenium dioxide. On the other hand, benzoselenadiazoles were synthesised by heating a mixture of *o*-phenylenediamine and selenium dioxide at 75 °C for 30 min^50^. In our research, the reaction of **1a–f** with SeO_2_ was carried out in a trial for obtaining the expected fused 1,2,5-selenadiazolo[3,4-*d*]pyrimidine **5,** however, instead, the oxo-selaneylidene derivative **4** was obtained after only 3 min of heating of **1b** with SeO_2_ (Scheme 2). The structure of compound **4** was proved by ^1^H NMR, ^13^C NMR, mass spectra, and elemental analysis. While the fusion of corresponding diaminouracils **1a, c–f** with selenium dioxide (SeO_2_) in a few drops of DMF for 13–15 min afforded 4-alkyl- or 4-aryl-1,2,5-selenadiazolo[3,4-*d*]pyrimidine-5,7-(4*H*,6*H*)-diones **5a–e** in interesting yields. The ^1^H NMR of compound **4** confirmed the presence of the NH_2_-6 group at δ 7.73 ppm and the disappearance of δ value of NH_2_-5. Besides, its mass spectra showed M^+^ + 2 = 361 and M^+^ 359. Whereas the ^1^H NMR of compounds **5a–e** demonstrated unequivocally the disappearance of 5,6-diamino groups of uracils. (EDX) is used to analyse the elemental composition of solid surfaces by irradiating the surface with a high-energy beam of charged particles, which stimulates the X-ray emission of the excited particles, giving a distinctive energy signature to each element, like a fingerprint. The composition of selenium compounds can be confirmed *via* EDX analysis of the Se^0^ particles, as reported^51^. The EDX analysis of the Se^0^ particles for compounds **5c** and **5e** are shown in Figure S10 and Figure S12 in the Figure S10 and Supplementary File. The selenium particles showed characteristic absorption peaks at 1.4 (Se Lα peak), 11.4 KeV (Se Kα peak), and 12.5 KeV (Se Kβ peak). The band located on the left part of the spectrum at around 0.2 keV indicates the presence of carbon, while the peak located at 0.5 KeV indicates the presence of oxygen. Nitrogen peaks were also visible in the EDX spectra at around 0.3 keV. The absence of other characteristic peaks and the presence of a high amount of selenium in the spectra confirm the purity of selenium metal in the prepared samples. The presence of carbon, oxygen, and nitrogen in the samples confirms the stability of the composition of the prepared sample.

As illustrated in Scheme 3, cyclo-condensation of diaminouracil hydrochloride **6** with selenium dioxide was achieved in ethanol at room temperature in the presence of triethylamine as a basic medium to liberate the free diaminouracil, affording **7** in a good yield.

### Biological evaluation

#### In vitro cytotoxic activity against HepG2, A-549, and MCF-7 cell lines

The viability assay (MTT method) was used for screening of anti-proliferative activity of **3a–f**, **5a–e,** and **7** against three cell lines, including MCF-7 cells (human breast cancer cell line), HepG-2 cells (human hepatocellular carcinoma), and A-549 (lung carcinoma) using methotrexate and 5-fluorouracil as the reference drugs. Compounds **3a** and **5d** showed the highest activity in all three cell lines, comparable to methotrexate but less than 5-fluorouracil. According to their anti-proliferative activity, compounds can be classified into three categories: those with low activity, those with low to moderate activity, and those with moderate to high activity compared to methotrexate. Compounds **3c**, **3f**, **5e,** and **7** demonstrated low anti-proliferative activity. Compounds **3b**, **3e**, **5a,** and **5b** showed low to moderate activity, whereas compounds **3a**, **3d,** and **5d** exhibited moderate to high activity. Interestingly, compounds with scaffold I showed higher anti-proliferative activity than those with scaffold II. Scaffold I compounds were characterised by the uracil core attached to the 5,5-dimethyl cyclohexanone moiety through an NH linker and bearing an amino group at position 6. The activity of such compounds could be attributed to the larger number of hydrogen bond donors when compared to compounds with scaffold II. On the other hand, the activity of **5d** with scaffold II could be attributed to the nature of the substituent at *N*1 of the pyrimidine ring being a hydrophobic benzyl group that could be involved in hydrophobic interaction and π-π stacking. Generally, the isosteric substitution of oxo with thioxo at position 2 of the pyrimidine ring lowered the anti-proliferative activity in both scaffolds I and II compounds. Table 1 shows the anti-proliferative activity of compounds **3a–f**, **5a–e**, and **7** against HepG2, A-549, and MCF-7 cell lines compared to methotrexate and 5-fluorouracil.

**Table 1.** Anti-proliferative activity of compounds **3a–f**, **5a–e**, and **7** against HepG2, A-549, and MCF-7 cell lines.

Compounds number	IC_50_ values (mean ± SD) µM		
	HepG-2	A-549	MCF-7
**3a**	14.31 ± 0.83	30.74 ± 0.76	27.14 ± 1.91
**3b**	40.42 ± 1.25	36.42 ± 1.09	46.06 ± 1.63
**3c**	>100	>100	>100
**3d**	21.63 ± 0.76	33.32 ± 0.99	31.38 ± 1.39
**3e**	47.61 ± 1.39	44.02 ± 1.28	>100
**3f**	>100	>100	>100
**5a**	51.71 ± 2.09	58.04 ± 2.13	51.90 ± 1.48
**5b**	57.93 ± 2.43	68.74 ± 3.86	98.07 ± 3.45
**5c**	33.42 ± 1.09	42.52 ± 1.19	39.74 ± 1.32
**5d**	17.68 ± 0.81	26.77 ± 0.89	29.26 ± 0.86
**5e**	>100	>100	>100
**7**	>100	>100	>100
Methotrexate	21.95 ± 1.08	34.52 ± 2.19	29.47 ± 2.19
5-Fluorouracil	7.37 ± 0.29	12.31 ± 0.53	10.07 ± 0.44

#### *In vitro* cytotoxic activity against normal human WI-38 cell line

Compounds **3a** and **5d**, which demonstrated the highest activity in anti-proliferative screening and the enzyme inhibition assays, were investigated further for selective cytotoxicity using the caucasian fibroblast-like foetal lung cell line (WI-38), and doxorubicin was used as the reference compound. When compared to doxorubicin (IC_50_ = 19.62 ± 0.74 μM), the compounds **3a** (IC_50_ = 80.81 ± 3.04 μM) and **5d** (IC_50_ = 54.57 ± 2.05 μM) demonstrated high selective cytotoxicity. **3a** increased the half-minimal inhibitory concentration of normal human cells by four-fold. The IC_50_ values for compounds **3a** and **5d** against WI-38 in comparison to doxorubicin are shown in Table 2.

**Table 2.** The *in vitro* cytotoxic activity of compounds **3a** and **5d** against WI-38 cell lines compared to doxorubicin.

Compound	Cytotoxicity
	WI-38
	IC_50_ (mean ± SD)
	μM
**3a**	80.81 ± 3.04
**5d**	54.57 ± 2.05
Doxorubicin	19.62 ± 0.74

#### *In vitro* Topoisomerase II enzyme inhibition assay

Topoisomerase II inhibitors can cause DNA damage and halt the cell cycle^52^. **3a** and **5d**, which showed the greatest anti-proliferative activity in all three cell lines, were also tested for *in vitro* Topoisomerase II enzyme inhibitory activity at various concentrations with doxorubicin as a control. Compound **3a** strongly inhibited Topoisomerase II with an IC_50_ value of 4.48 ± 0.65 μM that is nearly comparable to doxorubicin (IC_50_ = 3.45 ± 0.21 μM), whereas compound **5d** had an IC_50_ value of 8.39 ± 1.07 μM, which is approximately twice as active as doxorubicin. The half-maximal *in vitro* inhibitory concentrations of Topoisomerase II enzyme for compounds **3a** and **5d** compared to reference doxorubicin are shown in Table 3.

**Table 3.** The *in vitro* inhibitory activity of Topoisomerase II and HSP90 enzymes for compounds **3a** and **5d**, compared to doxorubicin and geldanamycin, respectively.

Compound	Topoisomerase II	HSP90
	IC_50_ (mean ± SD)	IC_50_ (mean ± SD)
	μM	μM
**3a**	4.48 ± 0.65	1.78 ± 0.11
**5d**	8.39 ± 1.07	7.69 ± 0.46
Doxorubicin	3.45 ± 0.21	–
Geldanamycin	–	0.842 ± 0.05

#### *In vitro* HSP90 enzyme inhibition assay

As reported, cancer cell growth and proliferation are inhibited when HSP90 is inhibited^53^. **3a** and **5d**, which showed the greatest anti-proliferative activity in all three cell lines, were tested for *in vitro* HSP90 enzyme inhibitory activity at various concentrations with geldanamycin as a control. Compound **3a**, with IC_50_ value of 1.78 ± 0.11 μM, inhibited HSP90 more effectively than compound **5d** (IC_50_ = 7.69 ± 0.46 μM).Table 3 shows the half-maximal *in vitro* inhibitory concentrations of the HSP90 enzyme for compounds **3a** and **5d** compared to reference geldanamycin.

#### Flow cytometric analysis of cell-cycle distribution

**Figure 3.**
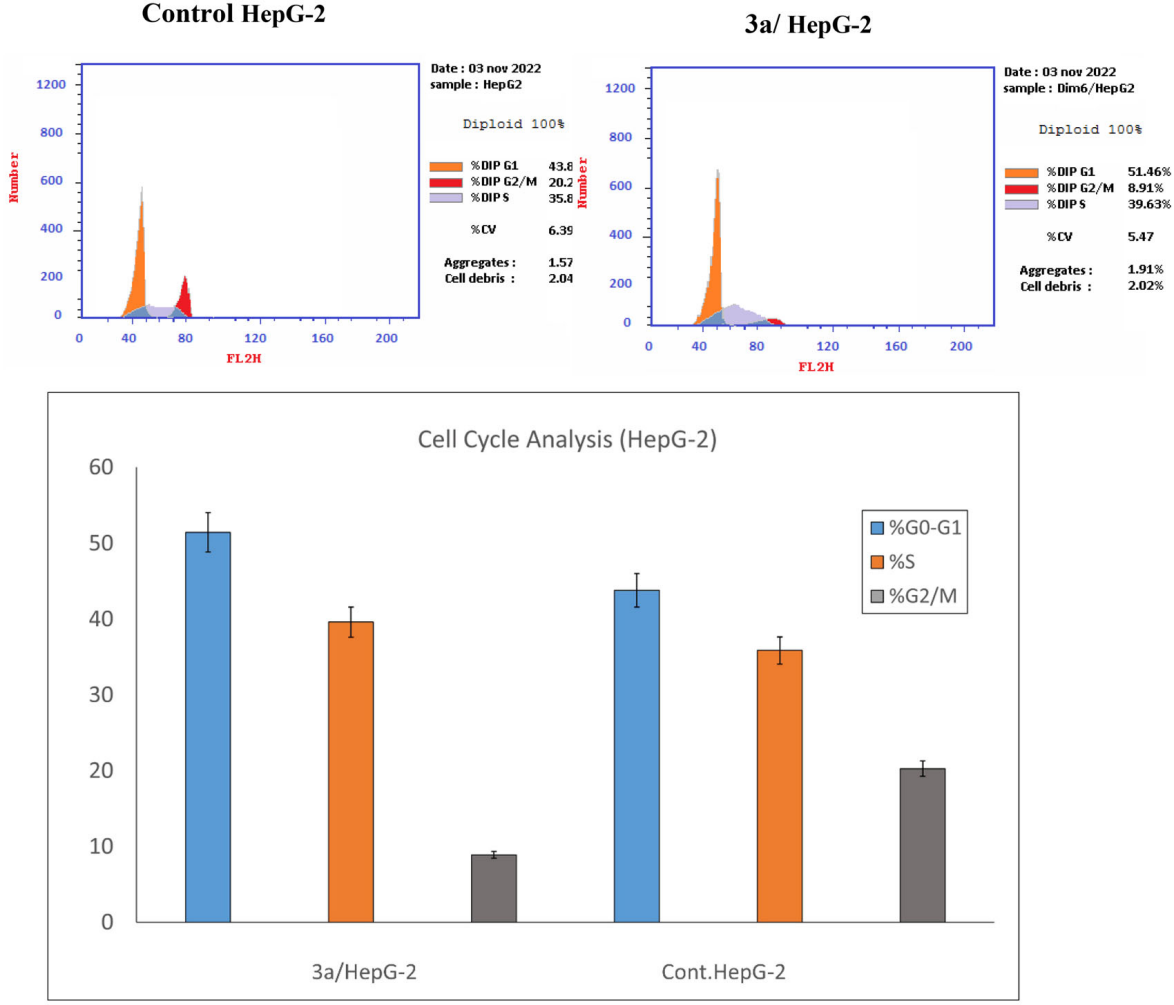
Effect of compound **3a** on HepG-2 cell cycle distribution using propidium iodide flow-cytometry assay.

**3a** demonstrated the most potent anti-proliferative activity against HepG-2 cell lines and selective cytotoxicity, as well as strong inhibition of Topoisomerase II and HSP90 enzymes. Thus, it was studied further to determine the mechanism of its cell growth inhibition. The standard PI flow cytometry assay was used to investigate cell cycle distribution and apoptosis induction in HepG-2 cells. As shown in Figure 3, treatment with **2a** at a concentration of 14.31 μM increased the proportion of arrested cells in the G1-S phase from 43.82% (±2.61) in untreated cells to 51.46% (±1.86) in treated cells. **3a** halts the cell cycle of HepG-2 at the G1-S phase.

#### Cellular apoptosis analysis

**Figure 4.**
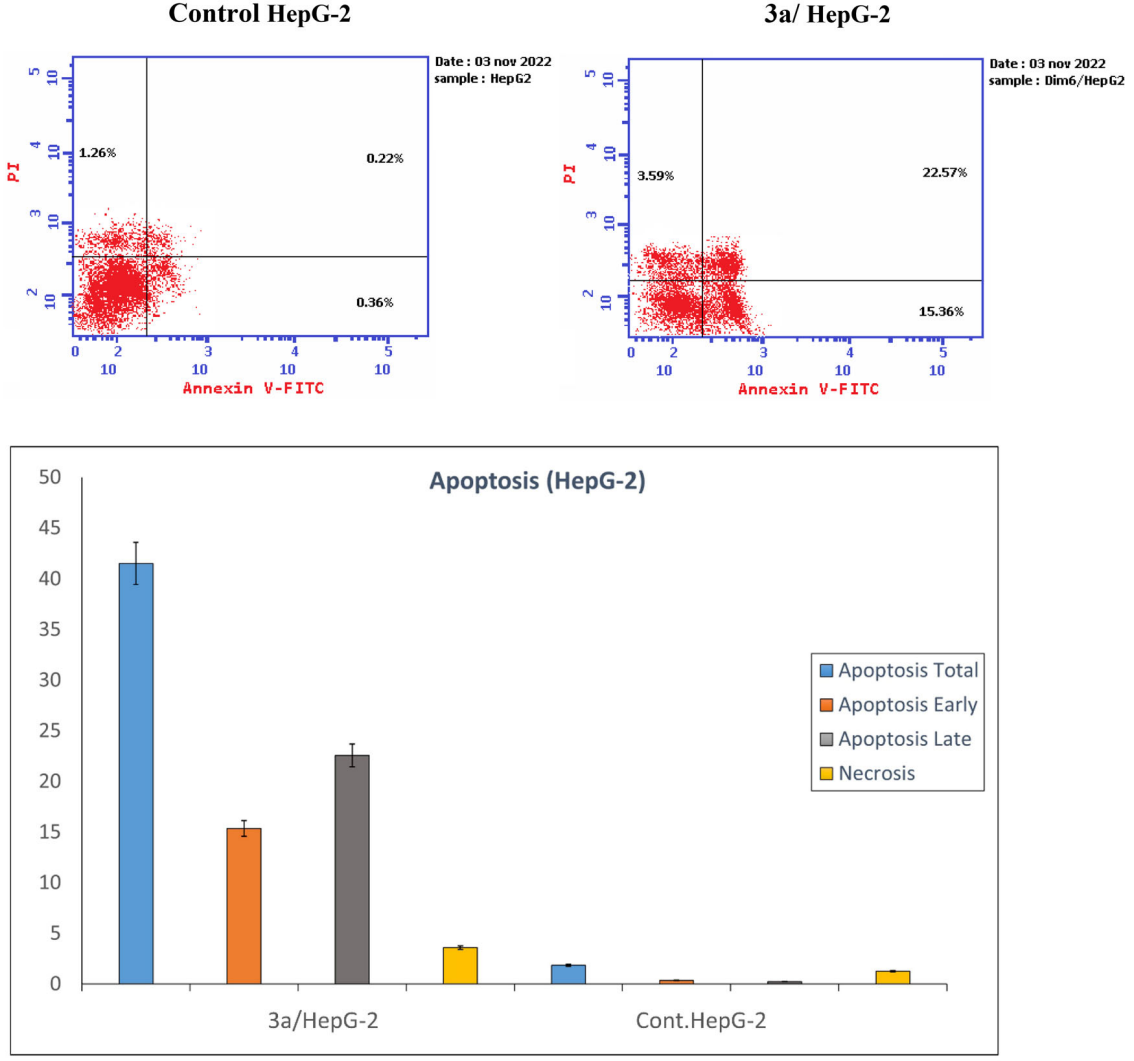
Apoptosis induced by **3a** on HepG-2 cells using Annexin FITC/PI dual staining assay.

Using HepG-2 cells and the Annexin FITC/PI dual-labelling technique, **3a**-induced apoptosis was investigated. According to Figure 4, the upper left quadrat Q1 indicates the ratio of necrotic cells, the upper right quadrat Q2 represents the ratio of late apoptotic cells, while the lower left quadrat Q3 and the bottom right quadrat Q4 demonstrate the ratio of early apoptotic cells. Treatment with **3a** at a concentration of 14.31 μM increased the total apoptotic cells from 0.58% in untreated cells to 37.93%, which was about 65 times higher than in control HepG-2 cells.

#### Estimation of Topoisomerase II, HSP90, and caspase-3 gene expression

**Figure 5.**
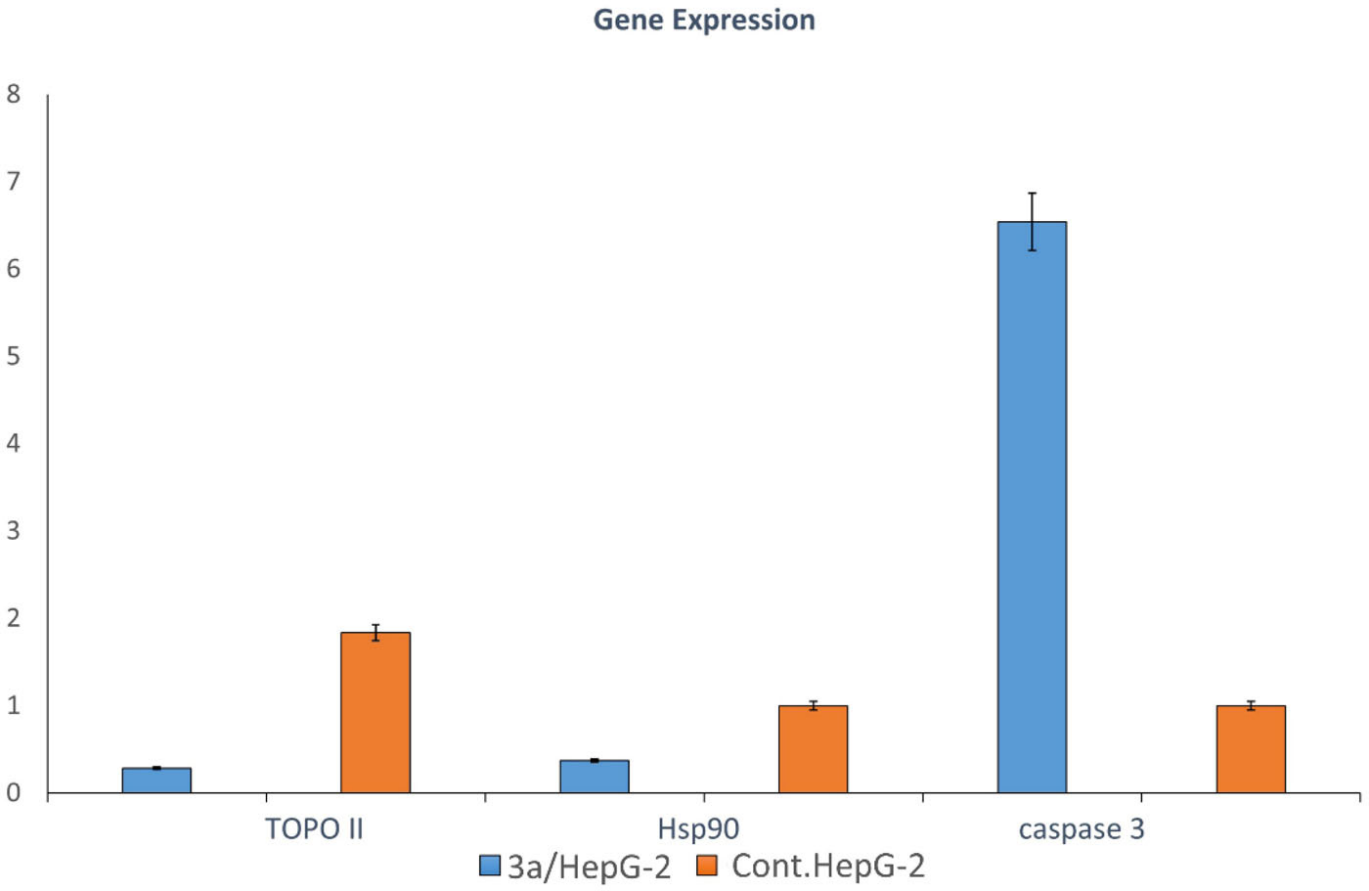
The effect of **3a** on the expression of genes encoding caspase-3, Topoisomerase *II*, and HSP90 in the supernatant of HepG-2 cell lines.

A caspase family is a group of cysteine proteases that activates apoptosis through receptor or mitochondrial-dependent cell death. Caspase-3 activation catalyses the cleavage of key cellular proteins, leading to both mitochondrial-dependent and non-dependent cell death. Caspase-3 is also associated with cell rupture and the formation of apoptotic bodies^54^. The effect of **3a** on caspase-3 gene expression was studied in HepG-2 cell lines. The caspase-3 gene was upregulated by **3a** when compared to control cells and control. This suggests that **3a** has the ability to induce apoptosis through both mitochondrial-dependent and non-dependent cell death. The gene that encodes DNA Topoisomerase II controls the topology of the enzyme during DNA transcription. This gene is a target for anti-proliferative agents by altering enzyme activity. In the present work, the effect of **3a** on the expression of the gene encoding Topoisomerase II in the supernatant of HepG-2 cell lines was studied. The results suggest that **3a** was able to downregulate Topoisomerase II gene expression when compared to control cells and control. Furthermore, the HSP90 encoding gene interferes with the proper folding of specified protein targets and interferes with ATP binding^55^. As well, the expression of gene encoding HSP90 was studied in the supernatant of HepG-2 cell lines, and **3a** downregulated HSP90 gene expression compared to control cells and control. Figure 5 shows the effect of **3a** on the expression of genes encoding caspase-3, Topoisomerase II, and HSP90 in the supernatant of HepG-2 cell lines.

### In silico studies

#### Molecular docking simulation

**Figure 6.**
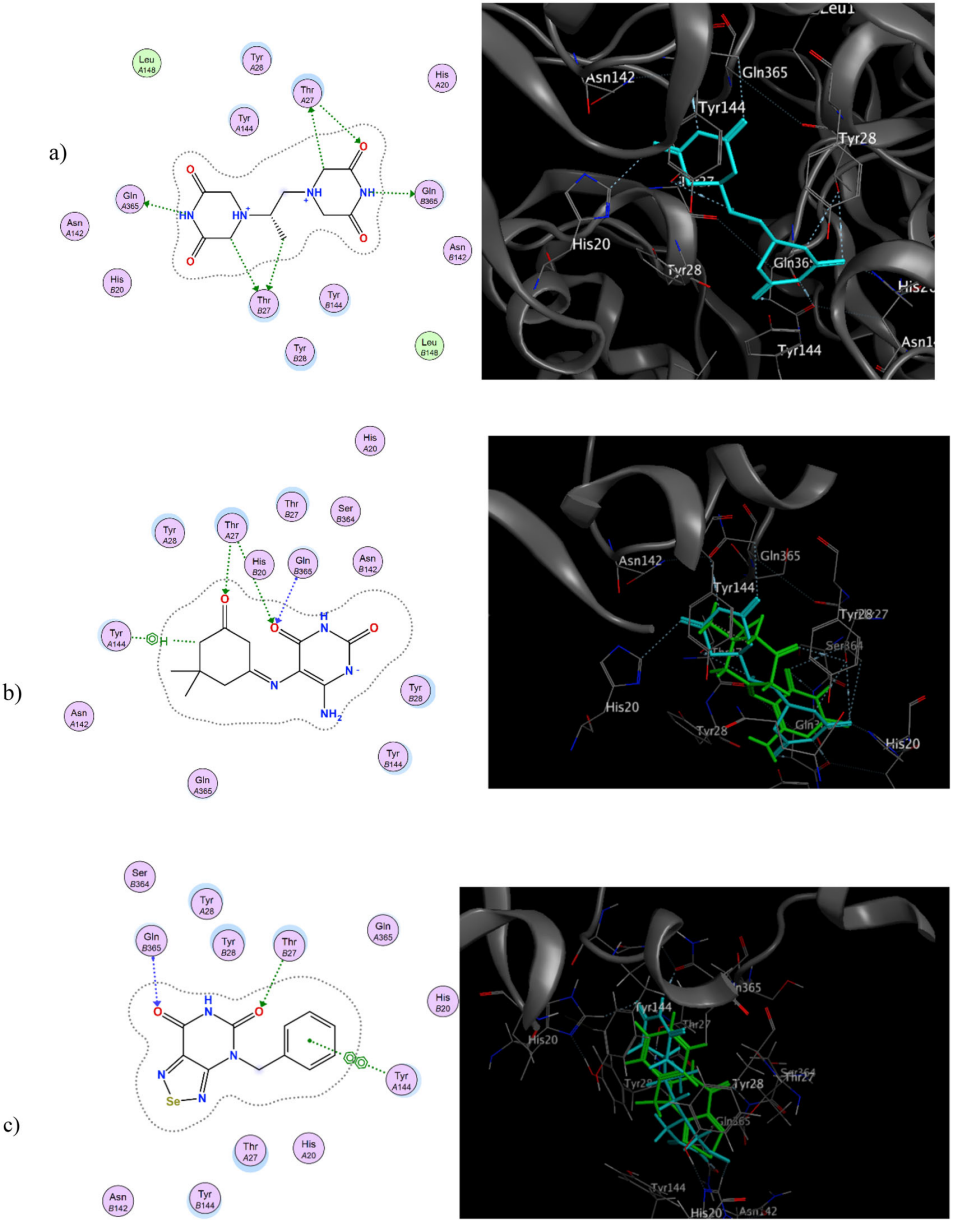
2D & 3D representation of dexrazoxane (a), 2D representation of **3a** & 3D representation of overlay view of **3a** and dexrazoxane (b), 2D representation of **5d** & 3D representation of overlay view of **5d** and dexrazoxane (c) at the binding site of Topoisomerase II (1QZR).

As stated, the Topoisomerase II enzyme is one of the key enzymes for cancer cell growth by enabling the coiling and detangling of DNA, making Topoisomerase II a key target for designing potent anti-proliferative agents^56^. Topoisomerase II has three catalytic domains: an N-terminal region (ATP-binding), a DNA binding, and a cleavage domain. Dexrazoxane, a non-competitive Topoisomerase II inhibitor, binds at its ATP binding site, which is composed of several key amino acids Tyr28, Thr27, and Gln365 (PDB ID:1QZR)^42^. The newly synthesised compounds **3a–f**, **5a–e,** and **7** were docked into the ATP binding site of Topoisomerase *II*. Docking poses with high energy scores were recorded. In comparison to dexrazoxane (-7.73 kcal/mol), **3a** and **5d** showed energy scores of −7.84 and −6.94 kcal/mol, respectively. **3a** formed two *H*-bond interactions at key amino acid at the ATP binding site Thr27, a stacking interaction at Tyr144, and an additional *H*-bond interaction at Gln365. Also, **5d** formed an *H*-bond interaction with key amino acid Thr27 at the ATP binding site of the Topoisomerase II enzyme. Figure 6 shows a 2D & 3D representation of dexrazoxane**, 3a,** and **5d** at the binding site of Topoisomerase II (1QZR).

**Figure 7.**
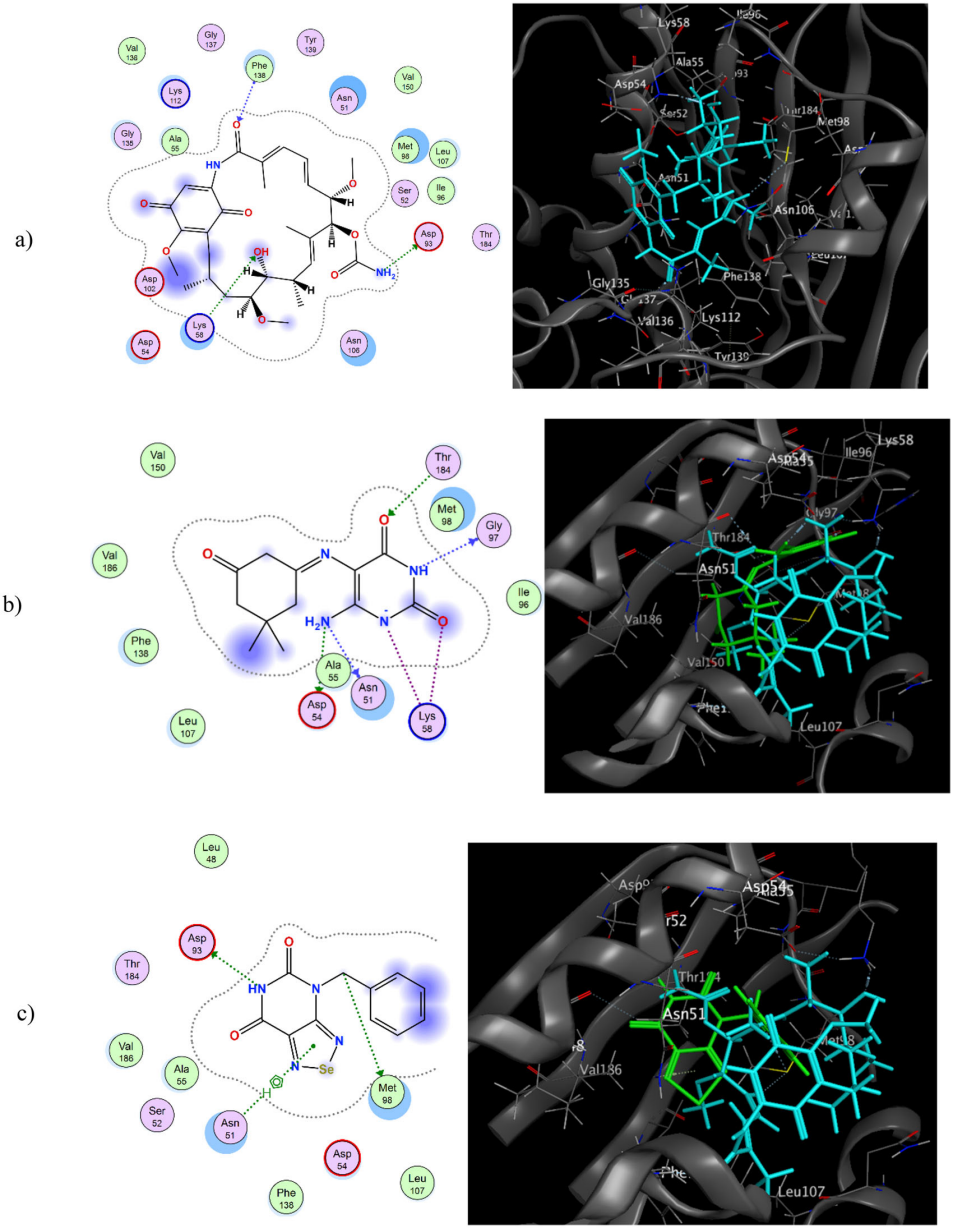
2D & 3D representation of geldanamycin (a), 2D representation of **3a** & 3D representation of overlay view of **3a** and geldanamycin (b), 2D representation of **5d** & 3D representation of overlay view of **5d** and geldanamycin (c) at the binding site of HSP90 (1YET).

As mentioned, HSP90 is a promising target for designing anti-proliferative agents. Inhibition of HSP90 results in the degradation of oncogenic proteins, which is crucial for cancer cell progression, preventing their aggregation and ATP-dependent refolding^57^. HSP90 consists of three main domains; the *N*-terminal domain (ATP binding pocket), the Middle domain, and the *C*-terminal domain. HSP90 has a helical structure with a hydrophobic and polar pocket at the top and a more hydrophobic pocket at the bottom. Asp93 and Phe138 are key amino acids at the HSP90 binding site^43^. Geldanamycin is a non-specific kinase inhibitor that can bind at the *N*-terminal domain of HSP90, forming an *H*-bond interaction with key amino acids Asp93, Lys112, and Phe138. Also, it forms hydrophobic and Vander-Waals interactions. The newly synthesised compounds **3a–f**, **5a–e,** and **7** were docked at the HSP90 geldanamycin binding domain (PDB ID:1YET)^43^. Docking poses with high energy scores were recorded. **3a** and **5d** showed energy scores of −11.51 and −10.81 k.cal/mol, respectively, compared to geldanamycin (-11.29 k.cal/mol). **3a** adopted a similar interaction mode to geldanamycin, forming *H*-bond interactions at key amino acids Asp93 and Asn51 at its amino group in position-6 of the pyrimidine ring. It also formed a hydrophobic interaction at Phe138. It formed two additional *H*-bond interactions at Lys59, Gly97, and Thr164. On the other hand, **5d** formed an *H*-bond interaction at Asp93 with an additional *H*-bond interaction at Met96, as well as an attacking interaction at Asn51. Figure 7 depicts a 2D & 3D representation of geldanamycin**, 3a,** and **5d** at the binding site of HSP90 (1YET). Table 4 shows the docking scores of compounds **3a–f**, **5a–e,** and **7** compared to co-crystalized native ligands, dexrazoxane and geldanamycin, at binding sites of Topoisomerase II and HSP90 enzymes, respectively.

**Table 4.** Docking results for the prepared compounds at Topoisomerase II (1QZR) and HSP90 (1YET) binding sites.

Compound	Docking score (kcal/mol)	
	Topoisomerase II binding site (1QZR)	HSP90 binding site (1YET)
**3a**	−7.84	−11.51
**3b**	−5.25	−10.27
**3c**	−6.09	−9.66
**3d**	−6.26	−10.60
**3e**	−5.38	−10.34
**3f**	−6.93	−9.84
**5a**	−5.65	−9.20
**5b**	−6.10	−8.58
**5c**	−6.32	−8.69
**5d**	−6.94	−10.81
**5e**	−5.86	−8.68
**7**	−6.48	−7.77
Dexrazoxane	−7.73	–
Geldanamycin	–	−11.29

#### Molecular modelling

**Figure 8.**
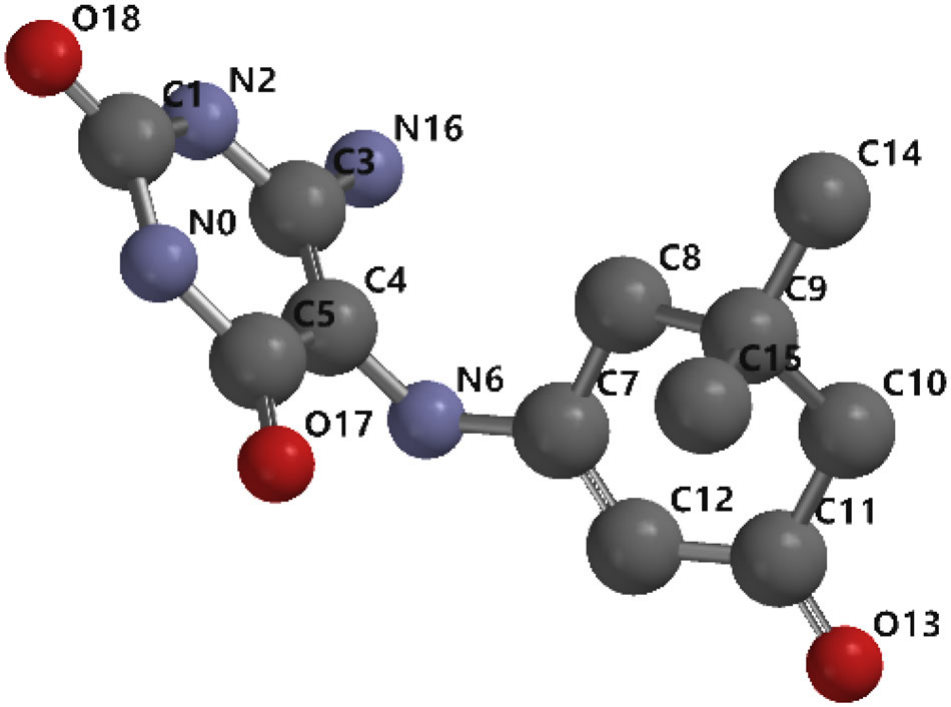
The geometry-optimised structure of **3a** compound.

The molecular structure of **3a** was geometrically optimised using the SPARTAN '18 program, as shown in Figure 8. The optimised parameters of bond lengths and bond angles were calculated by DFT/B3LYP with a 6–31 G* basis set, as shown in Table 5.

**Table 5.** Optimised geometrical parameters for the structure of **3a** computed at B3LYP/6-31G*.

Bond length	Values (Å)	Bond angle	Value (o)
(C1,O18)	1.216	(O18,C1,N2)	121.89
(C5,O17)	1.222	(C1,N2,C3)	124.56
(C11,O13)	1.227	(N2,C3,N16)	116.74
(C1,N0)	1.378	(N16,C3,C4)	123.06
(C1,N2)	1.401	(C4,C5,O17)	125.54
(C3,N16)	1.371	(C5,N0,C1)	127.71
(C4,N6)	1.422	(C3,C4,N6)	119.6
(C3,N2)	1.379	(C5,C4,N6)	120.09
(C5,N0)	1.416	(C4,N6,C7)	123.35
(C7,N6)	1.398	(C7,C8,C9)	113.65
(C3,C4)	1.374	(C8,C9,C10)	108.58
(C5,C4)	1.452	(C8,C9,C15)	110.08
(C7,C8)	1.509	(C8,C9,C14)	108.94
(C8,C9)	1.548	(C15,C9,C14)	108.94
(C9,C10)	1.543	(C15,C9,C10)	110.49
(C10,C11)	1.529	(C14,C9,C10)	109.79
(C11,C12)	1.458	(C9,C10,C11)	114.21
(C12,C7)	1.358	(C10,C11,C12)	116.72
(C9,C14)	1.538	(C11,C12,C7)	122.64
(C9,C15)	1.543	(C8,C7,C12)	122.01
		(C10,C11,O13)	120.97
		(C12,C11,O13)	122.27

The common bond orbital investigation provides a proficient strategy for examining intra and intermolecular bonding, bond interaction, and charge exchange in the molecular system. The atomic charges of the considered atoms were calculated, as shown in Table 6. There are twelve carbon atoms, seven of which are positively charged, and the other is negatively charged. Four nitrogen atoms and three oxygen atoms were discovered to have a negative atomic charge.

**Table 6.** Mulliken atomic charges of **3a** calculated at DFT/B3LYP/6-31G* level of theory.

Atom	Mulliken charges	Atom	Mulliken charges	Atom	Mulliken charges
C1	+0.770	C10	−0.353	N2	−0.732
C3	+0.571	C11	+0.447	N6	−0.731
C4	+0.079	C12	−0.282	N16	−0.773
C5	+0.625	C14	−0.449	O13	−0.508
C7	+0.390	C15	−0.451	O17	−0.507
C8	−0.341	N0	−0.705	O18	−0.495
C9	+0.076				

##### HOMO-LUMO analysis

**Figure 9.**
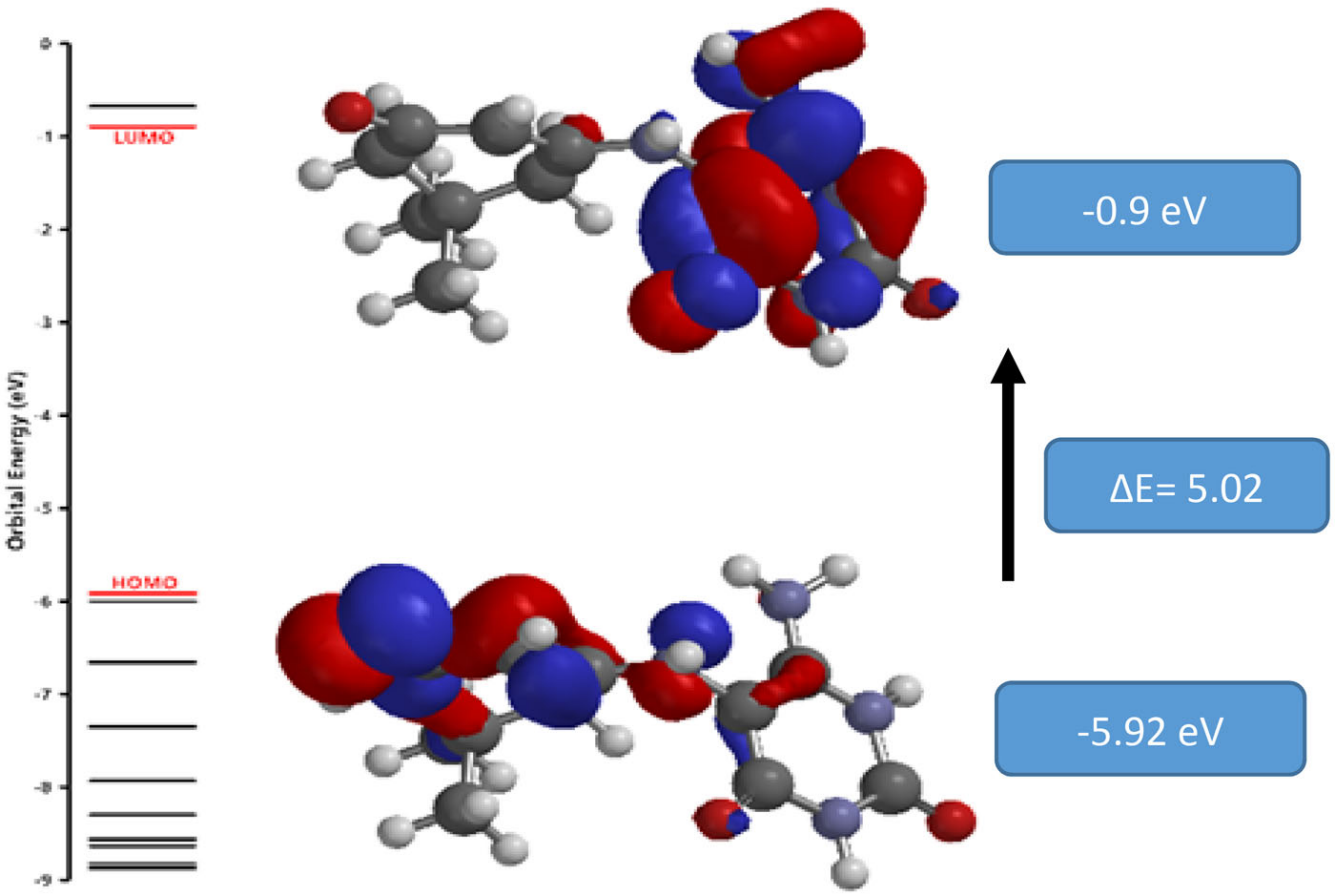
The atomic orbital compositions of the molecular orbital for **3a**.

The highest occupied molecular orbital (HOMO) and the lowest non-occupied molecular orbital (LUMO) are important parameters for understanding the kinetic stability of the molecule. The positive phase is represented in red, while the negative phase is shown in blue. The LUMO value was found to be −0.9 eV, whereas the obtained HOMO value was −5.92 eV, resulting in an energy gap of ΔE = 5.02 eV between the two occupied and unoccupied levels. Figure 9 illustrates the HOMO and LUMO of **3a** molecular orbitals.

By using the values of HOMO and LUMO, we can calculate some other parameters, like the ionisation potential (I), which is known as the required energy to remove an electron from the outer shell of a neutral atom or molecule. Also, electronegativity χ (χ = I + A/2), chemical potential μ (μ = -χ), chemical hardness η (η = I-A/2), and chemical softness S (S = 1/2η), as well as the global electrophilicity index ω (ω = μ2/2η), were calculated^58^. Global electrophilicity^59^ is a better descriptor of global chemical reactivity because it demonstrates energy stabilisation when the system acquires an additional electronic charge from outside, as well as information about both electron transfer (chemical potential) and stability (hardness). All these calculated parameters are shown in Table 7.

**Table 7.** molecular orbital energies, HOMO-LUMO gap, and global reactivity descriptors for **3a**, calculated at B3LYP/6-31G* level.

Molecular parameters	Value
Total energy (Kcal/mol)	−572,049
Dipole moment (Deby)	7.41
E LUMO (eV)	−0.9
E HOMO (eV)	−5.92
ΔEg_ap_ (eV)	5.02
Ionisation potential, IP (eV)	5.920
Electron affinity, EA (eV)	0.900
Electronegativity, (χ)	3.410
Chemical potential, (μ)	−3.410
Hardness, (η) (eV)	2.510
Softness, (S) (eV)-1	0.199
Electrophilicity, (ω)	2.316

##### Molecular electrostatic potential (MEP)

**Figure 10.**
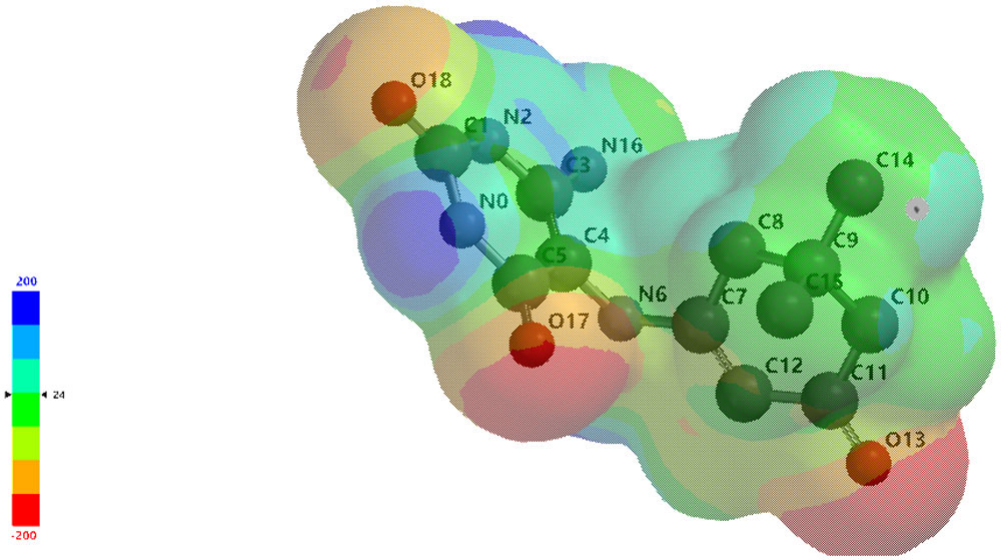
The total electron density surface was mapped with a molecular electrostatic potential MEP plot for Sparfloxacin calculated using the B3LYP/6-31G* method.

The molecular electrostatic potential (MEP) refers to electron density in the molecule that is important for understanding electrophilic and nucleophilic reaction sites as well as hydrogen bonding interactions in the system^37^. MEP was calculated using B3LYP/6–31G* method, and the obtained results show and predict the reactive sites of the electrophilic or nucleophilic molecule. The electrostatic potential surface appears in different colours, red, blue, and green, corresponding to the most negative, positive, and zero electrostatic potential regions, respectively. The negative electrostatic potential represents proton attraction by the molecule’s aggregate electron density (red colour), whereas the positive electrostatic potential corresponds to proton repulsion by the atomic nuclei (blue colour). Figure 10 depicts the total electron density surface mapped with the molecular electrostatic potential MEP plot (solid and mesh views) for the calculated compound **3a**.

#### *In silico* physicochemical properties, drug-likeness data, and ADME profile of most active compounds 3a and 5d compared to 5-fluorouracil

In order to be considered a potential drug candidate, compounds should have specific characteristics, such as physicochemical properties, pharmacokinetics or pharmacodynamics, and drug-likeness. As a result, physicochemical properties, drug-likeness, and ADME properties for the most active compounds **3a** and **5d** were studied compared to the antitumor drug (5-fluorouracil) using the Swiss-ADME online tool (www.SwissADME.ch)^60^.

##### Physicochemical properties and drug-likeness

Several parameters, including structural, molecular, and physicochemical properties expressed in Lipinski’s rule, should be considered. Lipinski’s rule (rule of five) represents broad and general guidelines for orally bioavailable drug candidates, which include the following parameters: molecular weight (M. wt. ≤ 500 g/mol), number of hydrogen bond acceptors ≤ 5, number of hydrogen bond donors ≤ 10, and partition coefficient (log P) ≤5^61^. Topological polar surface area (TPSA), which is another important physicochemical property that needs to be studied for drug candidates, is used to express the surface associated with polar atoms, ideally TPSA ≤ 160^62^. Similar to 5-fluorouracil, both compounds **3a** and **5d** showed no violation of Lipinskìs rule, with molecular weights ranging from 305.19 g/mol to 354.40 g/mol. Furthermore, compounds **3a** and **5d** had acceptable partition coefficients and topological polar surface areas ranging from −0.61 to 2.09 and 54.86 to 109.98, respectively, whereas log p and TPSA of 5-fluorouracil were −0.73 and 65.72, respectively. Moreover, both compounds had 2 to 4 rotatable bonds, 2 to 3 hydrogen bond acceptors, and 1 to 3 hydrogen bond donors following Lipinskìs rule of five (Table 8).

**Table 8.** Physicochemical properties and drug likeness for compounds **3a** and **5d**.

	M.W (g/mol)	ilog P	TPSA (Å)	HBA	HBD	RB	Lipinski violations
**3a**	354.40	2.09	109.98	3	3	4	0
**5d**	305.19	−0.61	54.86	2	1	2	0
5-FU	130.08	−0.73	65.72	3	2	0	0

##### The ADME profile

ADME properties, including absorption, distribution, metabolism, and excretion, were studied using an *in silico* tool (Table 9). As for solubility, compounds **3a** and **5d** were soluble with log S −1.91 to −2.05, while 5-fluorouracil had log S of −0.01 with high solubility. Based on the applied method, Insoluble <-10 < Poorly<-6 < Moderately<-4 < Soluble<-2 < very < 0 < Highly^63^. Additionally, both compounds showed high gastrointestinal absorption as designated by the white-boiled egg model, indicating their ability to be easily absorbed through the gut wall^64^. Furthermore, both compounds demonstrated no ability to penetrate the blood-brain barrier and hence no CNS side effects, as indicated by the yolk of the boiled egg model, which was similar to 5-fluorouracil^64^. Moreover, compounds **3a** and **5d** were suggested to have no side effects of potential liver toxicity, as indicated by their non-inhibitory abilities of Cytochrome P450 (CYP2D6, Cyp2C9, Cyp2C19, and Cyp1A2).

**Table 9.** The ADME study results for compounds **3a** and **5d**.

Cpd. no.	Solubility	BBB permeant	GI absorption	Cytochrome P450			
				(CYP inhibitor)			
				CYP2D6 inhibitor	CYP2C9 inhibitor	CYP2C19 inhibitor	CYP1A2 inhibitor
**3a**	−1.91	No	High	No			
**5d**	−2.05	No	High	No			
5-FU	−0.01	No	High	No			

##### The toxicity properties

The toxicity and carcinogenicity of compounds were studied using Admet lab0.2 online source^65^. Compound **3a** showed improved properties, including no possible carcinogenicity or mutagenicity as indicated by the Ames test, no acute oral toxicity, no respiratory, no skin toxicity, and no ability to block HERG enzyme that indicated no potential cardiovascular side effects^66^. On the other hand, 5-fluorouracil showed potential acute oral toxicity in the rat model (Table 10). The physicochemical properties, drug-likeness, ADME study results, and *in silico* toxicity properties for all compounds **3a–f**, **5a–e,** and **7** are provided in the Supplementary Data, Tables S1, S2, and S3, respectively.

**Table 10.** *In silico* toxicity properties for compounds **3a** and **5d**.

Cpd. no.	Carcinogenicity	Ames test	Rat oral toxicity	Skin toxicity	HERG Blocker	Respiratory toxicity
**3a**	−ve	−ve	−ve	−ve	−ve	−ve
**5d**	+ve	−ve	−ve	−ve	−ve	−ve
5-FU	−ve	−ve	+ve	−ve	−ve	−ve

## Conclusion

To conclude, 3-oxocyclohex-1-enyl-5-aminopyrimidines (**3a–f**) and fused 1,2,5-selenadiazolopyrimidines (**5a–e** and **7**) were synthesised as cyclohexanone-pyrimidine hybrids (Scaffold I) and selenadiazole-pyrimidine hybrids (Scaffold II). In comparison to methotrexate and 5-fluorouracil, the compounds showed low, low to moderate, and moderate to high anti-proliferative activities against three cell lines, human hepatocellular carcinoma (HepG-2), lung carcinoma (A-549), and human breast cancer cell line (MCF-7). Scaffold I compounds were more active anti-proliferative, where **3a** showing the highest activity against HepG-2 (IC_50_ = 14.31 ± 0.83 µM), A-549 (IC_50_ = 30.74 ± 0.76 µM), and MCF-7 (IC_50_ = 27.14 ± 1.91 µM). Compound **3a** demonstrated selective cytotoxicity, with an IC_50_ of 80.81 ± 3.04 µM against a normal Caucasian fibroblast-like foetal lung cell line (WI-38). **3a** exhibited its anti-proliferative activity possibly through dual inhibition of two enzymes crucial for cancer cell growth, Topoisomerase II and HSP90, where **3a** was able to strongly inhibit both enzymes *in vitro* Topoisomerase II (IC_50_ = 4.48 ± 0.65 µM) and HSP90 (IC_50_ = 1.78 ± 0.11 µM). The molecular modelling simulation also revealed that **3a** had the ability to bind at both Topoisomerase II and HSP90 enzyme binding sites in an inhibitory mode. Additionally, **3a** was capable of increasing total apoptotic cells 65 times more than control Hep-G2 cells and stopping the cell cycle at the G1-S phase. Furthermore, the gene expression studies of **3a** in the supernatant of HepG-2 cells showed that it was capable of downregulating both Topoisomerase II and HSP90 encoding genes while upregulating caspase-3 gene expression, indicating that its apoptotic effect is possibly due to both mitochondrial and non-mitochondrial influences. Furthermore, **3a** was geometrically optimised and investigated using density functional theory (DFT) with the B3LYP method of calculation and basis set (6-31 G*). Moreover, *in silico* studies for compound **3a** demonstrated a good oral bioavailability profile with no side effects to the CNS, liver, or cardiovascular system. As well, compound **3a** was non-mutagenic and non-carcinogenic, with good physicochemical properties that complied with Lipinski’s rule and were comparable to 5-fluorouracil.

## Supplementary Material

Supplemental Material
