# Supplementary material for: Synthesis, DFT calculations, and anti-proliferative evaluation of pyrimidine and selenadiazolopyrimidine derivatives as dual Topoisomerase II and HSP90 inhibitors
Source: J Enzyme Inhib Med Chem. 2023 Apr 10;38(1):2198163. doi: 10.1080/14756366.2023.2198163 (PMC10088926; doi:10.1080/14756366.2023.2198163)
Supplement: Supplemental Material [file IENZ_A_2198163_SM6327.pdf]

Charts supporting information

Synthesis, DFT calculations, and anti-proliferative evaluation of pyrimidine and selenadiazolopyrimidine derivatives as dual Topoisomerase II and HSP90 inhibitors

Samar El-Kalyoubi[1*], Samiha A. El-Sebaey[2*], A. M. Rashad[3,4], Hanan A. AL-Ghulikah[5], Mostafa M. Ghorab[6*], Sherin M. Elfeky[7]

[1]Department of Pharmaceutical Organic Chemistry, Faculty of Pharmacy, Port Said University, 42511 Port Said, Egypt.
[2]Department of Pharmaceutical Organic Chemistry, Faculty of Pharmacy (Girls), Al-Azhar University, Youssef Abbas Street, Nasr City, Cairo, Egypt
[3]Accelerator and Ion Sources Department, Nuclear Research Center, Atomic Energy Authority, Egypt.
[4]Central Lab for Elemental and Isotopic Analysis, NRC, Atomic Energy Authority, Egypt.
[5]Department of Chemistry, College of Science, Princess Nourah Bint Abdulrahman University, P.O. Box 84428, Riyadh 11671, Saudi Arabia
[6]Department of Drug Radiation Research, National Centre for Radiation Research and Technology (NCRRT), Egyptian Atomic Energy Authority (EAEA), Cairo 11765, Egypt
[7]Department of Pharmaceutical Organic Chemistry, Faculty of Pharmacy, Mansoura University, Mansoura 355516, Egypt.

*Corresponding authors:
1- Mostafa .M. Ghorab; mmsghorab@yahoo.com; Tel.: +20 2 2747413; fax: +20 2 2749298.
2- Samar El-Kalyoubi; S.elkalyoubi@pharm.psu.edu.eg; s.elkalyoubi@hotmail.com; https://orcid.org/0000-0002-4648-5184
3- Samiha A. El-Sebaey; samiha.ali85@azhar.edu.eg; https://orcid.org/0000-0002-9928-9080

Table of Contents

	Figure & Table number	Page number
^{1}H NMR, ^{13}C NMR and Mass spectra of 6-amino-5-((5,5-dimethyl-3-oxocyclohex-1-en-1-yl)amino)pyrimidine-2,4(1*H*,3*H*)-dione **(3a)**	**Figure S1**	S3, S4
^{1}H NMR, ^{13}C NMR and Mass spectra of 6-amino-1-(2-chlorobenzyl)-5-((5,5-dimethyl-3-oxocyclohex-1-en-1-yl)amino)pyrimidine-2,4(1*H*,3*H*)-dione **(3b)**	**Figure S2**	S5
^{1}H NMR, ^{13}C NMR and Mass spectra of 6-amino-5-((5,5-dimethyl-3-oxocyclohex-1-en-1-yl)amino)-1-methyl-2-thioxo-2,3-dihydropyrimidin-4(1*H*)-one **(3c)**	**Figure S3**	S6
^{1}H NMR, ^{13}C NMR and Mass spectra of 6-amino-5-((5,5-dimethyl-3-oxocyclohex-1-en-1-yl)amino)-1-ethylpyrimidine-2,4(1*H*,3*H*)-dione **(3d)**	**Figure S4**	S7, S8
^{1}H NMR, ^{13}C NMR and Mass spectra of 6-amino-1-benzyl-5-((5,5-dimethyl-3-oxocyclohex-1-en-1-yl)amino)pyrimidine-2,4(1*H*,3*H*)-dione **(3e)**	**Figure S5**	S9
^{1}H NMR, ^{13}C NMR and Mass spectra of 6-amino-5-((5,5-dimethyl-3-oxocyclohex-1-en-1-yl)amino)-1-methylpyrimidine-2,4(1*H*,3*H*)-dione **(3f)**	**Figure S6**	S10
^{1}H NMR, ^{13}C NMR and Mass spectra of 6-amino-1-(2-chlorobenzyl)-5-((oxo-λ^4-selaneylidene)amino)pyrimidine-2,4(1*H*,3*H*)-dione **(4)**	**Figure S7**	S11
^{1}H NMR, ^{13}C NMR and Mass spectra of [1,2,5]selenadiazolo[3,4-*d*]pyrimidine-5,7(4*H*,6*H*)-dione **(5a)**	**Figure S8**	S12
^{1}H NMR, ^{13}C NMR and Mass spectra of 4-methyl-5-thioxo-5,6-dihydro-[1,2,5]selenadiazolo[3,4-*d*]pyrimidin-7(4*H*)-one **(5b)**	**Figure S9**	S13
^{1}H NMR, ^{13}C NMR, Mass and EDX spectra of 4-ethyl-[1,2,5]selenadiazolo[3,4-*d*]pyrimidine-5,7(4*H*,6*H*)-dione **(5c)**	**Figure S10**	S14, S15
^{1}H NMR, ^{13}C NMR and Mass spectra of 4-benzyl-[1,2,5]selenadiazolo[3,4-*d*]pyrimidine-5,7(4*H*,6*H*)-dione **(5d)**	**Figure S11**	S16
^{1}H NMR, ^{13}C NMR, Mass and EDX spectra of 4-methyl-[1,2,5]selenadiazolo[3,4-*d*]pyrimidine-5,7(4*H*,6*H*)-dione **(5e)**	**Figure S12**	S17, S18
^{1}H NMR, ^{13}C NMR and Mass spectra of 4,6-dimethyl-[1,2,5]selenadiazolo[3,4-*d*]pyrimidine-5,7(4*H*,6*H*)-dione **(7)**	**Figure S13**	S19
Physicochemical properties and drug-likeness for the all synthesized compounds	**Table S1**	S20
The ADME study results for the all synthesized compounds	**Table S2**	S20
In silico toxicity properties for the all synthesized compounds	**Table S3**	S21

Figure S1: ^{1}H NMR, ^{13}C NMR and Mass spectra of 6-amino-5-((5,5-dimethyl-3-oxocyclohex-1-en-1-yl)amino)pyrimidine-2,4(1*H*,3*H*)-dione **(3a)**

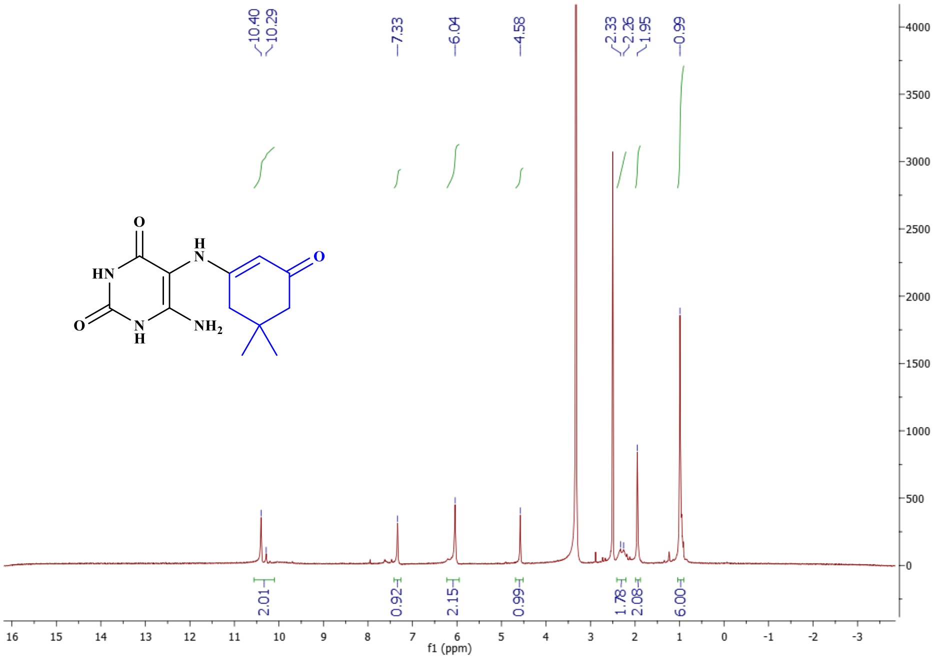

D$_2$O

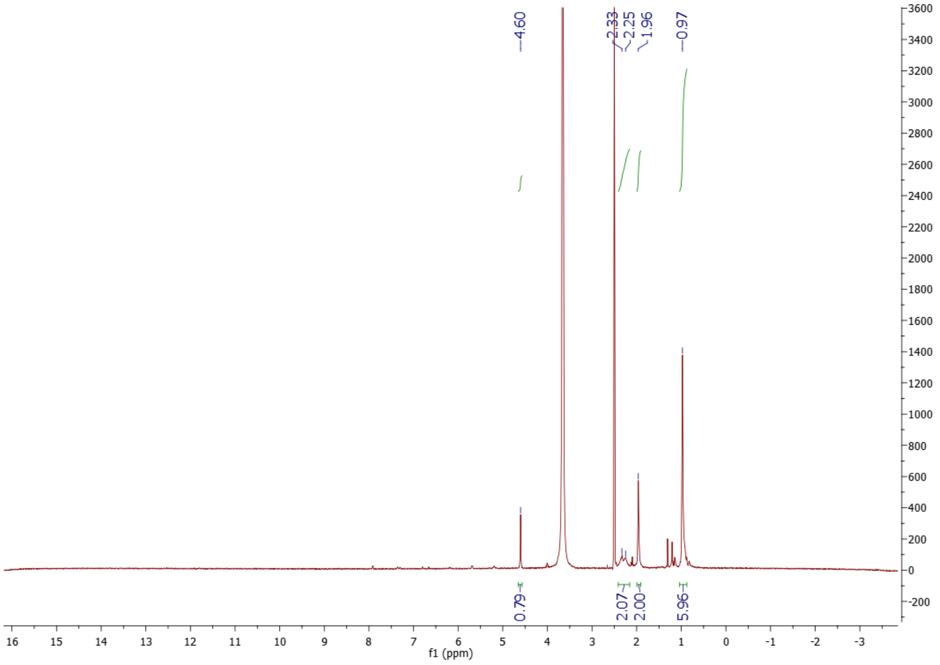

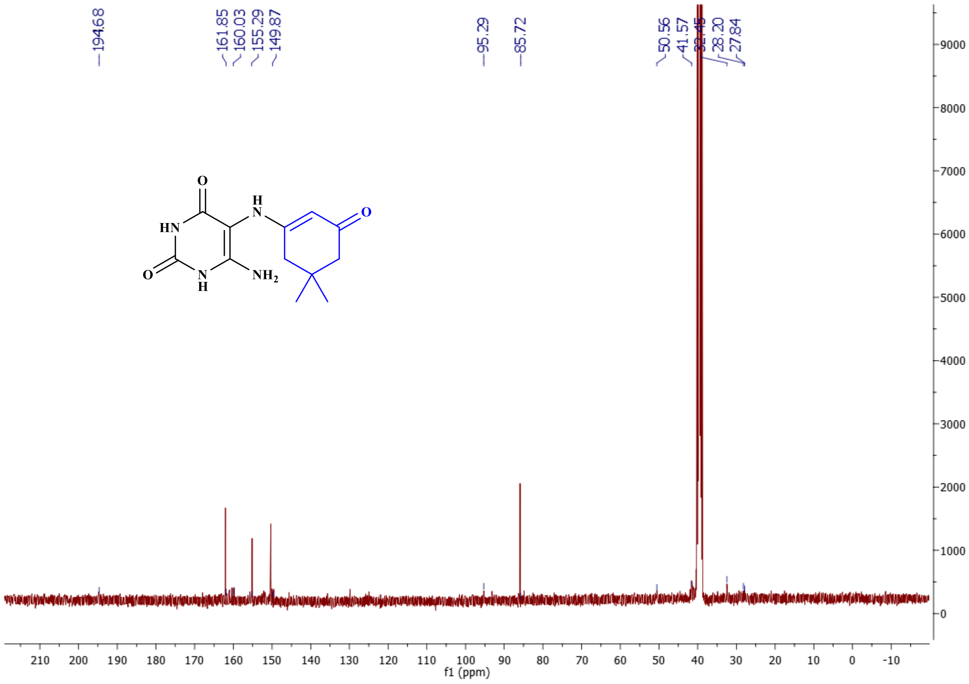
194.68
161.85
160.03
155.29
149.87
95.29
85.72
50.56
41.57
32.46
28.20
27.84
HN
H
N
NH2
O
f1 (ppm)

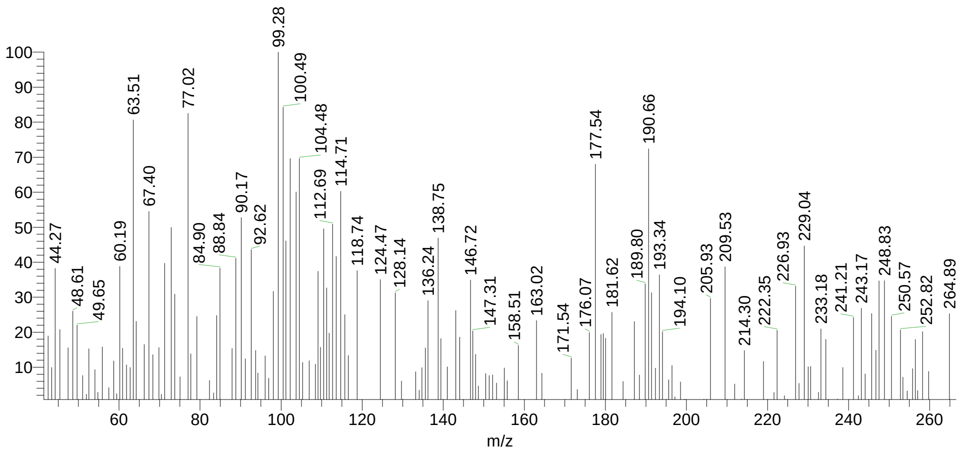
99.28
100.49
104.48
114.71
63.51
77.02
177.54
190.66
m/z

Figure S2: ^{1}H NMR, ^{13}C NMR and Mass spectra of 6-amino-1-(2-chlorobenzyl)-5-((5,5-dimethyl-3-oxocyclohex-1-en-1-yl)amino)pyrimidine-2,4(1*H*,3*H*)-dione **(3b).**

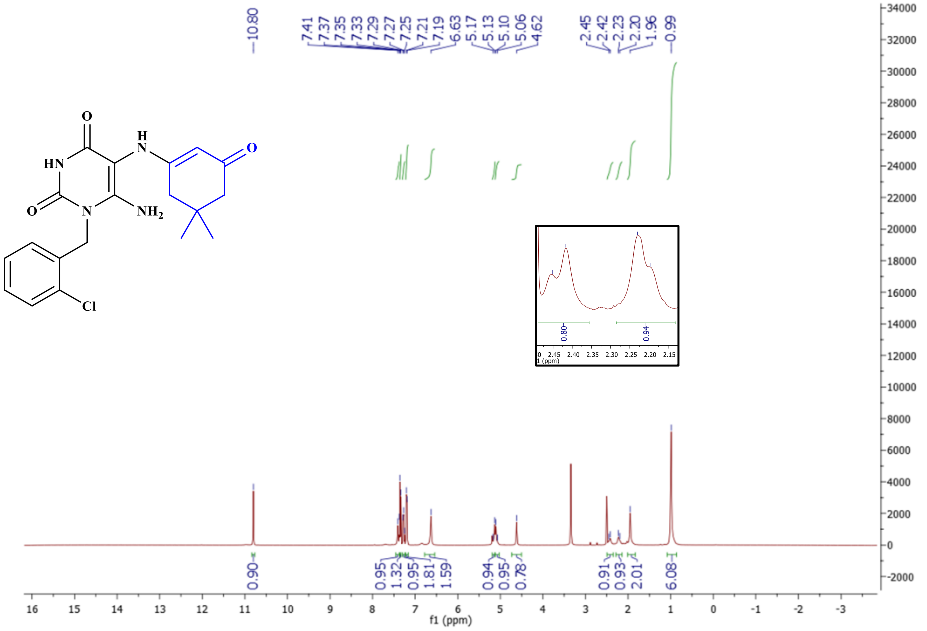

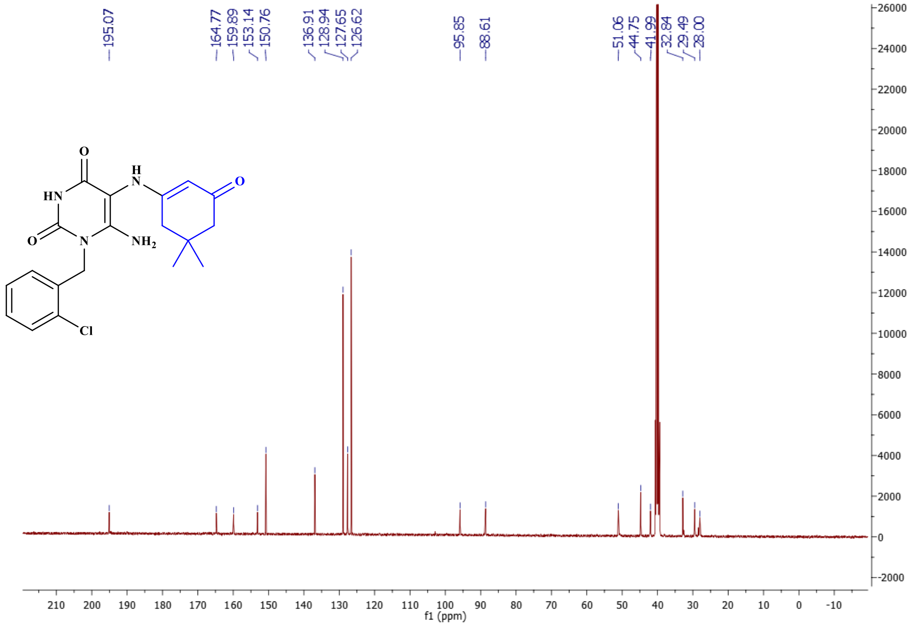

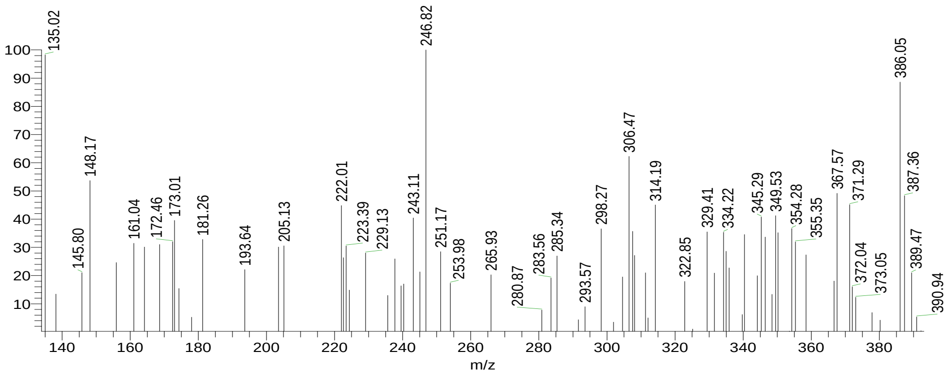

Figure S3: ^{1}H NMR, ^{13}C NMR and Mass spectra of 6-amino-5-((5,5-dimethyl-3-oxocyclohex-1-en-1-yl)amino)-1-methyl-2-thioxo-2,3-dihydropyrimidin-4(1*H*)-one **(3c).**

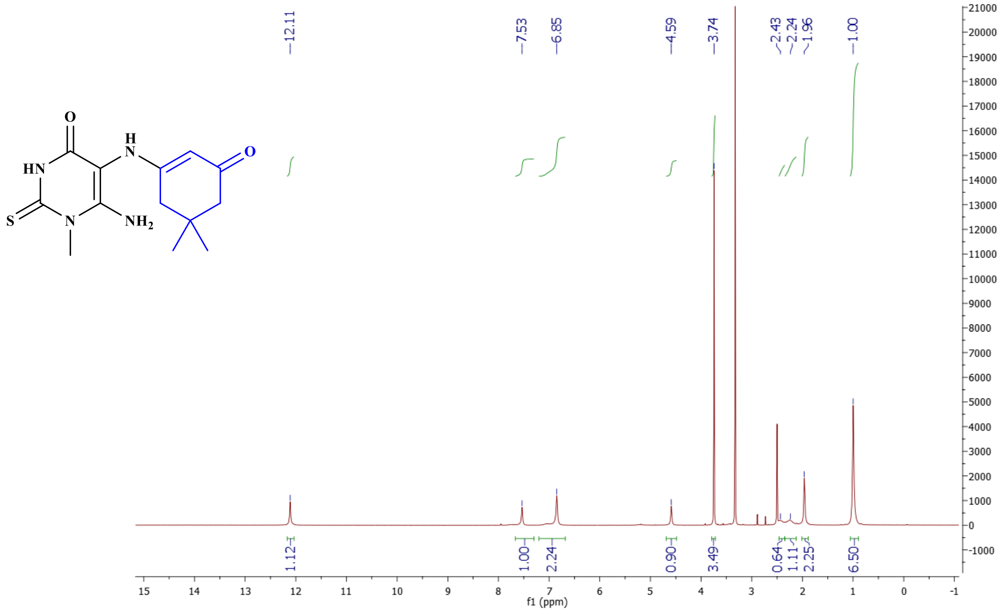

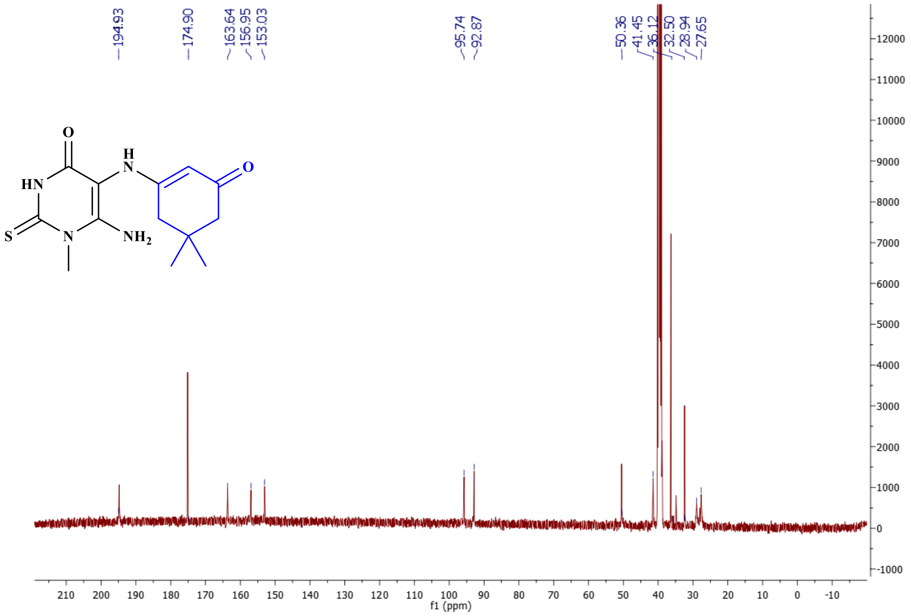

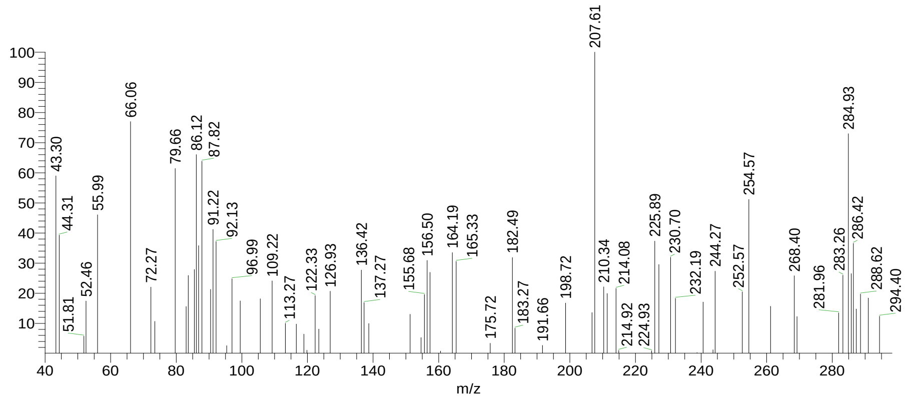

Figure S4: ^{1}H NMR, ^{13}C NMR and Mass spectra of 6-amino-5-((5,5-dimethyl-3-oxocyclohex-1-en-1-yl)amino)-1-ethylpyrimidine-2,4(1*H*,3*H*)-dione **(3d).**

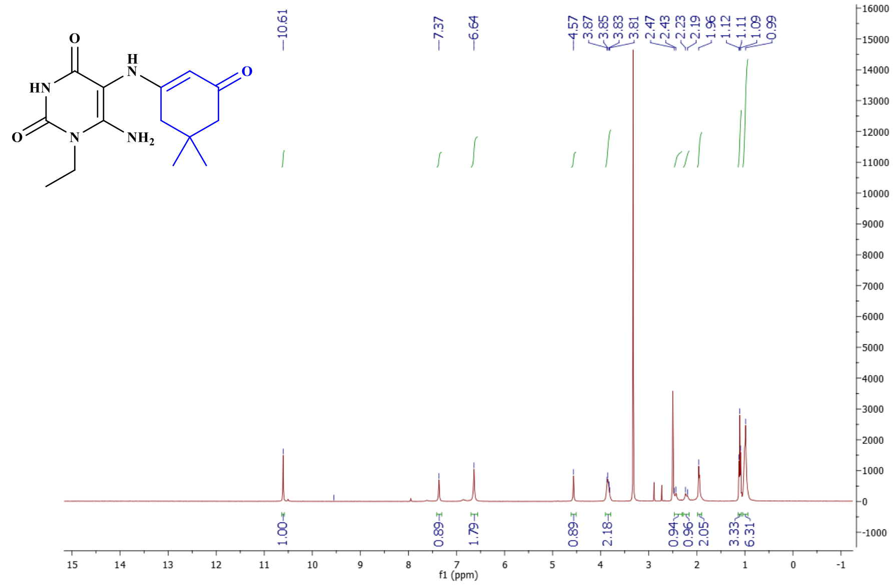

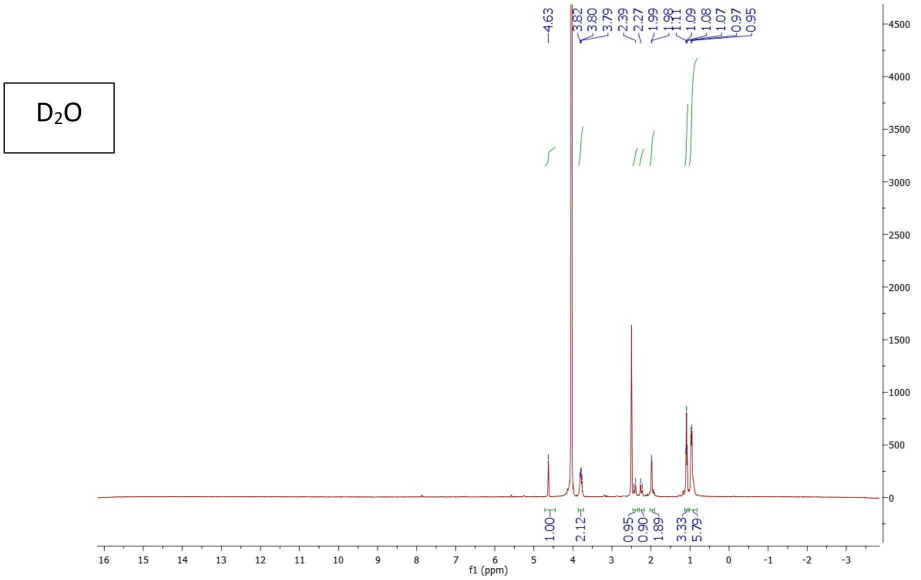

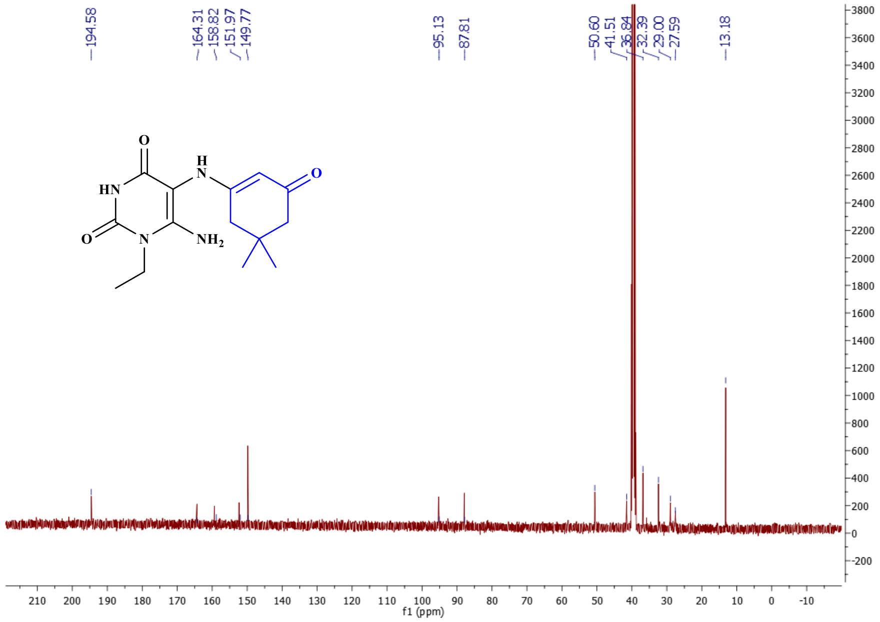
194.58
164.31
158.82
151.97
149.77
95.13
87.81
50.60
41.51
36.84
32.39
29.00
27.59
13.18
f1 (ppm)

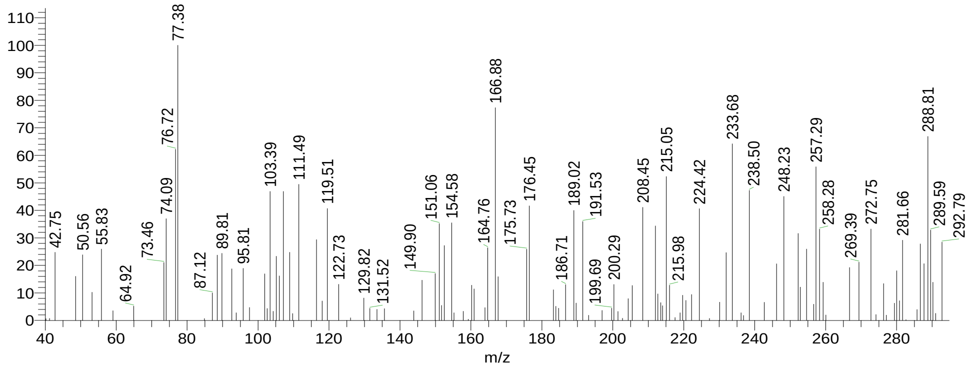
m/z

Figure S5: ^{1}H NMR, ^{13}C NMR and Mass spectra of 6-amino-1-benzyl-5-((5,5-dimethyl-3-oxocyclohex-1-en-1-yl)amino)pyrimidine-2,4(1*H*,3*H*)-dione **(3e).**

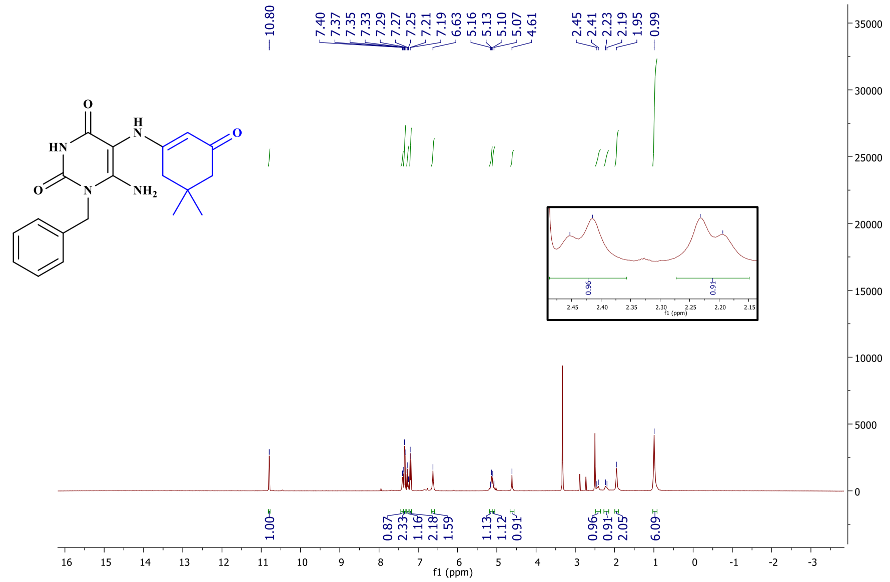

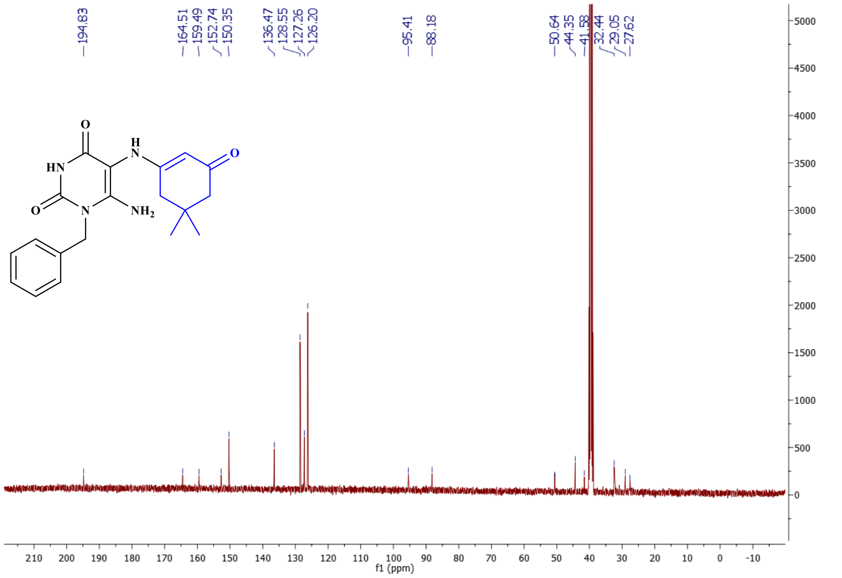

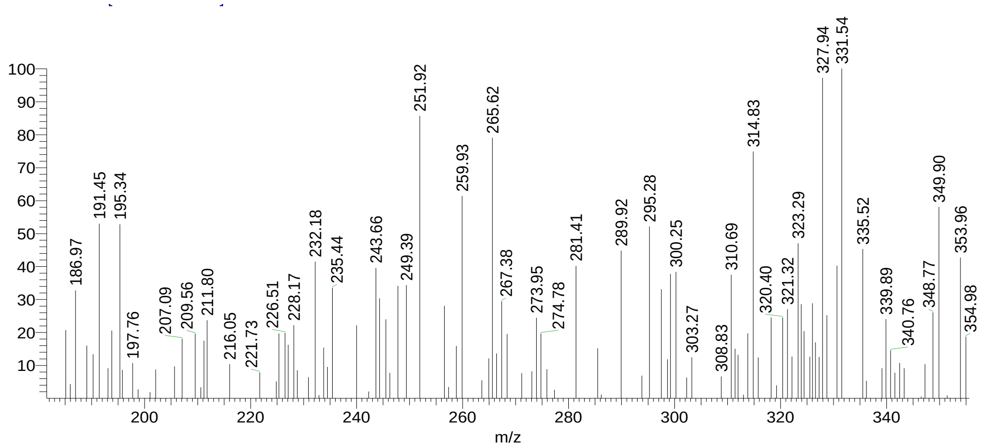

Figure S6: ^{1}H NMR, ^{13}C NMR and Mass spectra of 6-amino-5-((5,5-dimethyl-3-oxocyclohex-1-en-1-yl)amino)-1-methylpyrimidine-2,4(1*H*,3*H*)-dione **(3f).**

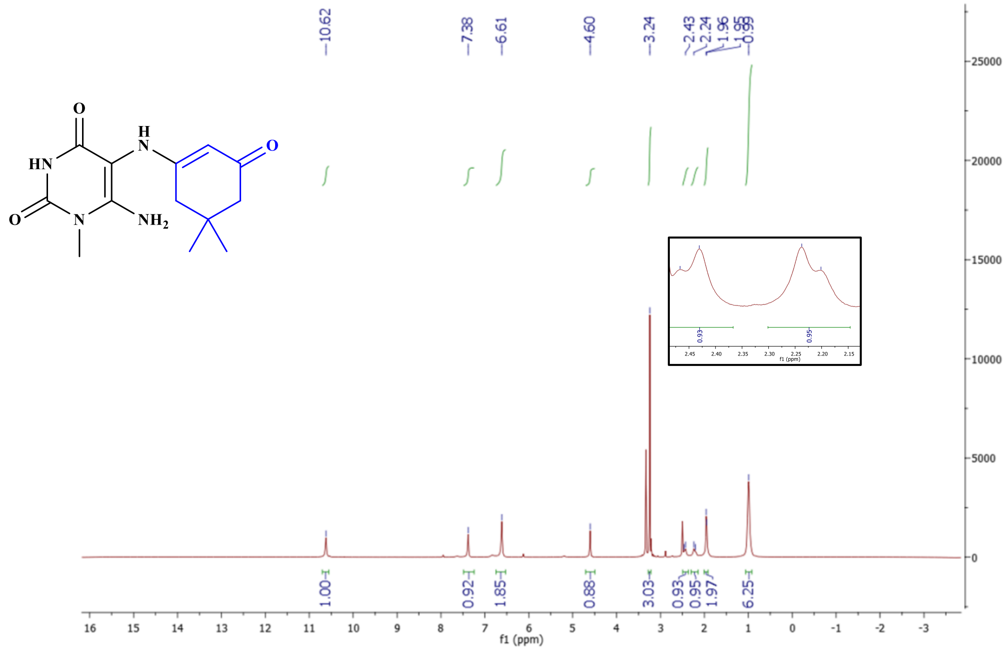

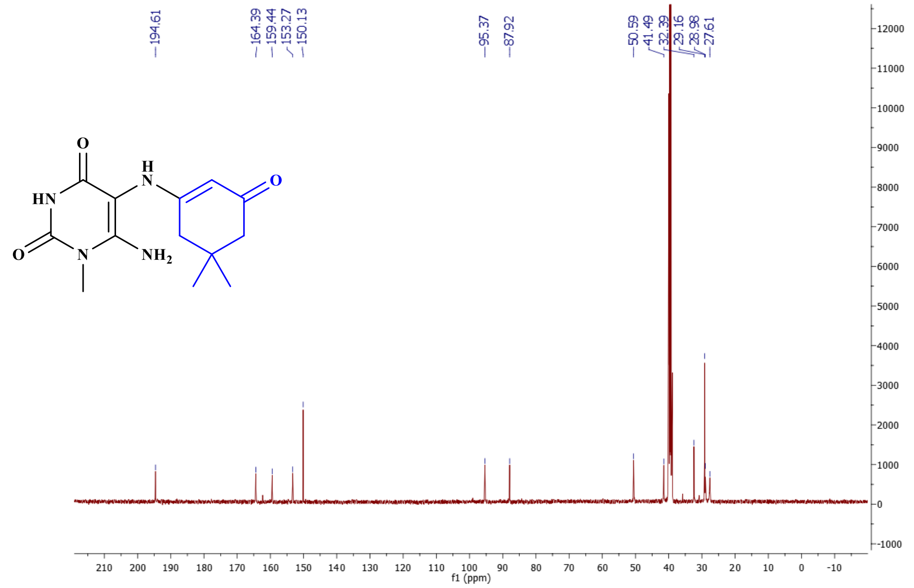

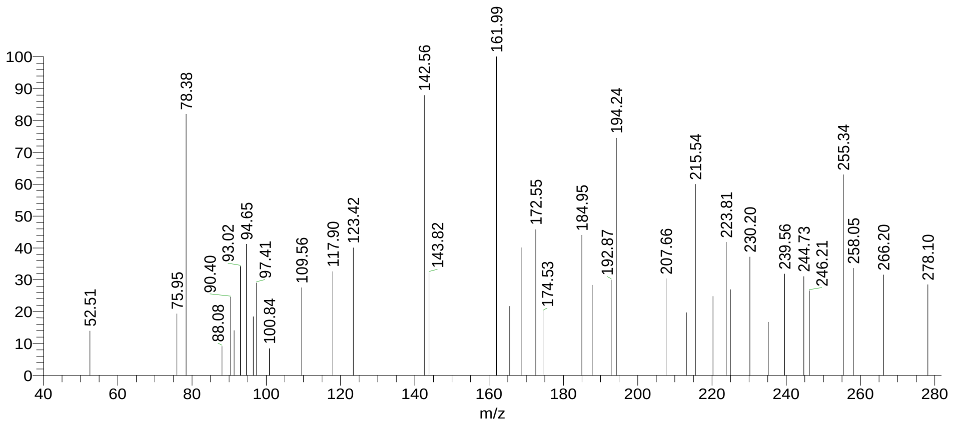

Figure S7: ^{1}H NMR, ^{13}C NMR and Mass spectra of 6-amino-1-(2-chlorobenzyl)-5-((oxo-λ^4-selaneylidene)amino)pyrimidine-2,4(1*H*,3*H*)-dione **(4).**

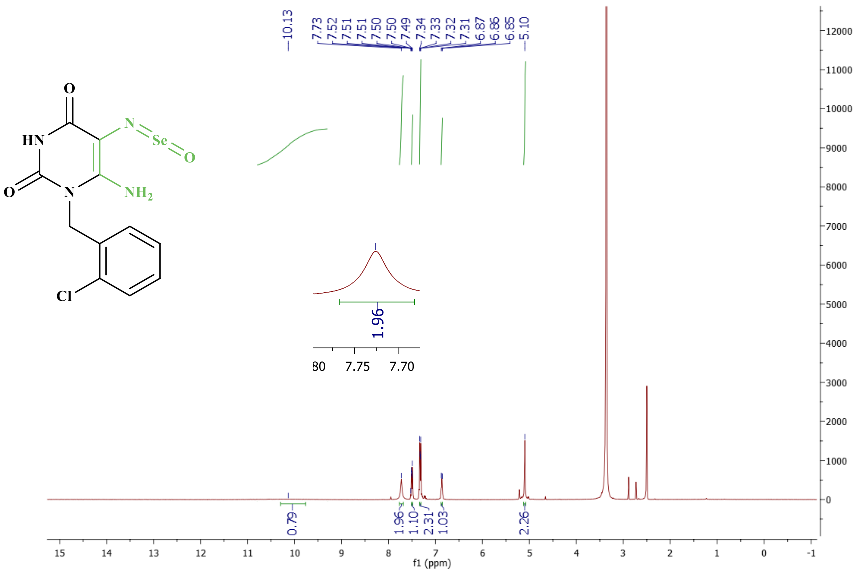

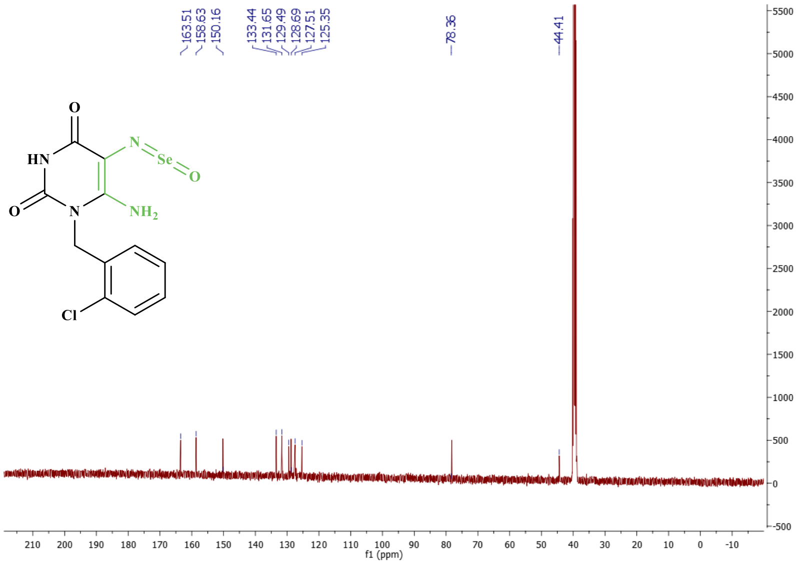

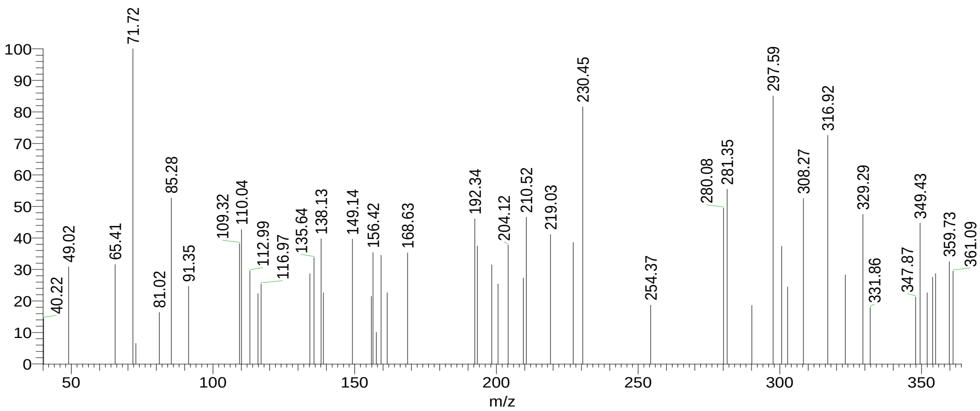

Figure S8: ^{1}H NMR, ^{13}C NMR and Mass spectra of [1,2,5]selenadiazolo[3,4-*d*]pyrimidine-5,7(4*H*,6*H*)-dione **(5a)**

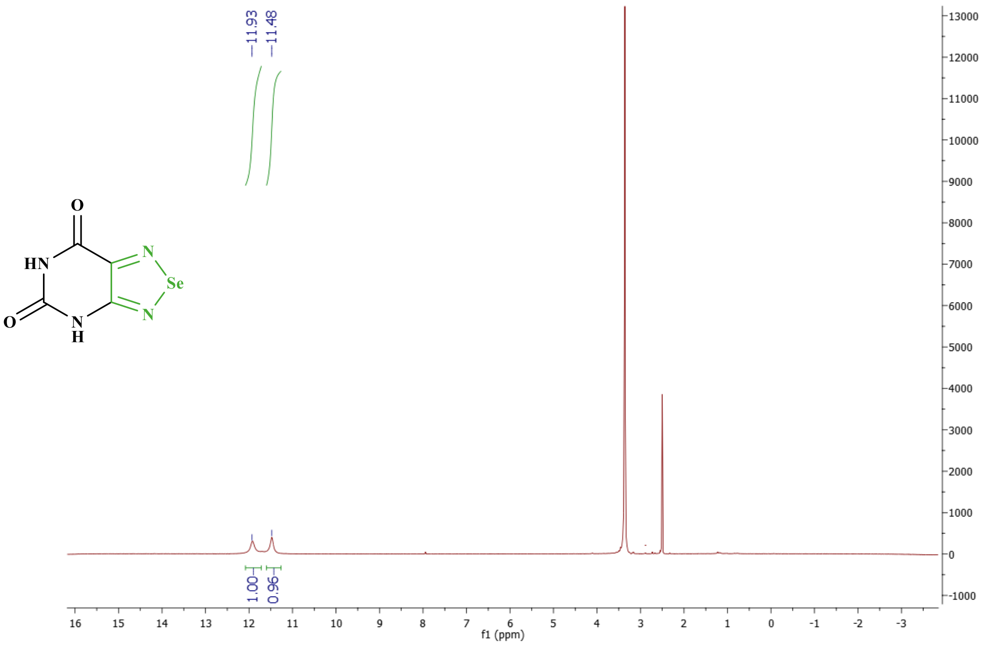

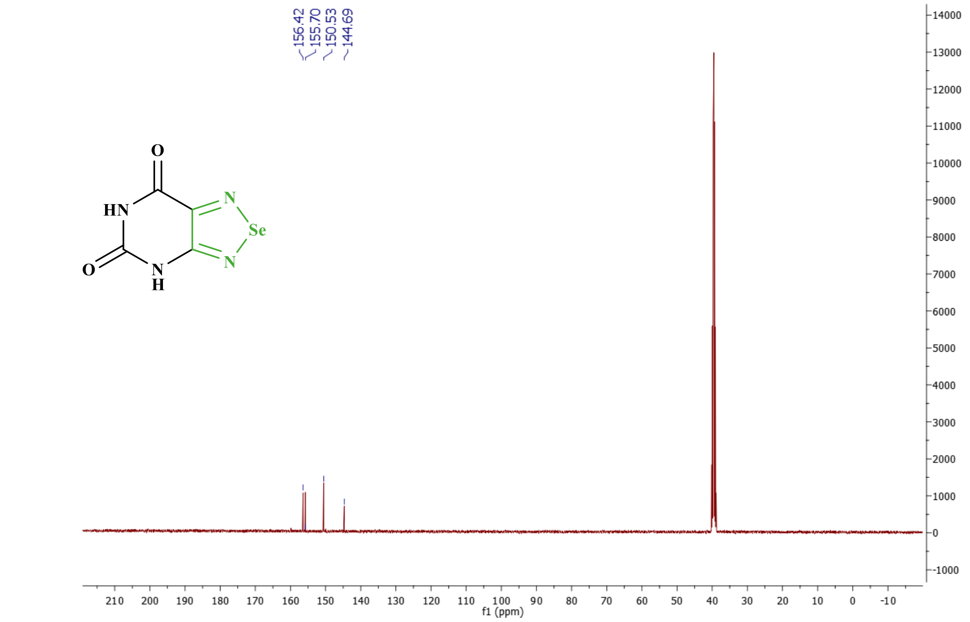

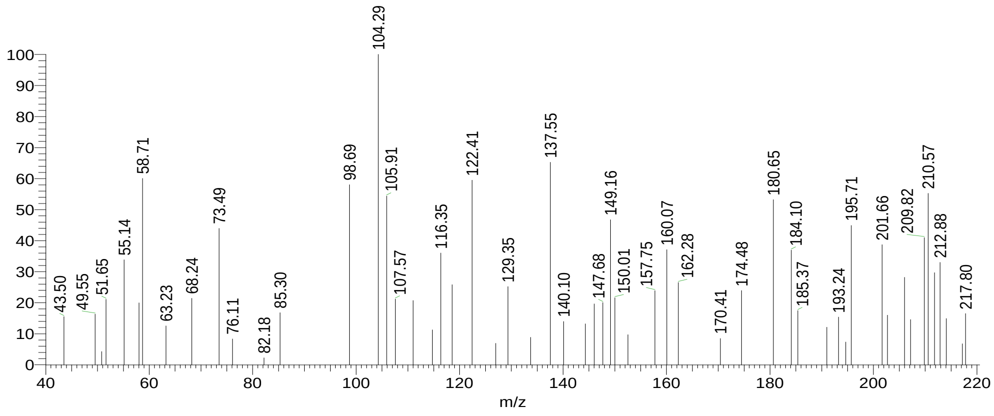

Figure S9: ^{1}H NMR, ^{13}C NMR and Mass spectra of 4-methyl-5-thioxo-5,6-dihydro-[1,2,5]selenadiazolo[3,4-*d*]pyrimidin-7(4*H*)-one **(5b).**

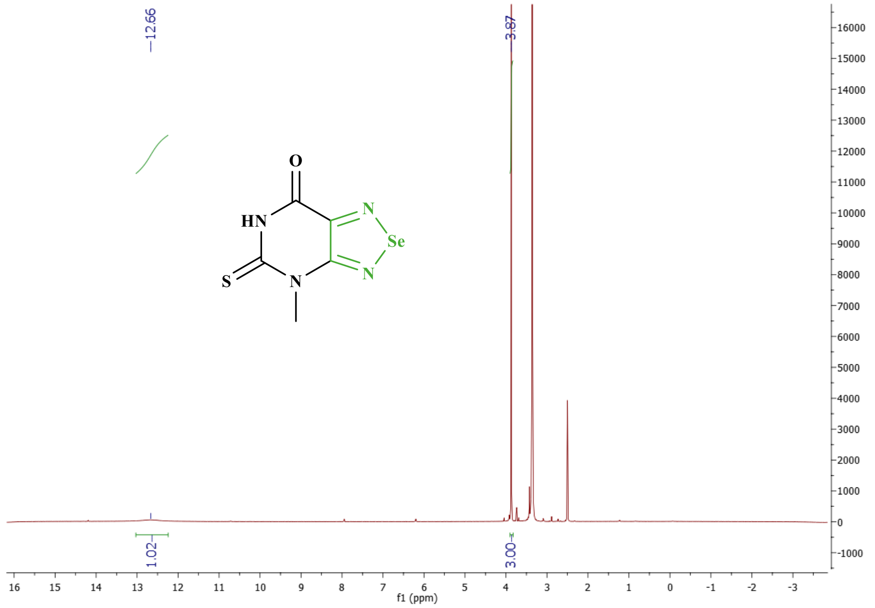

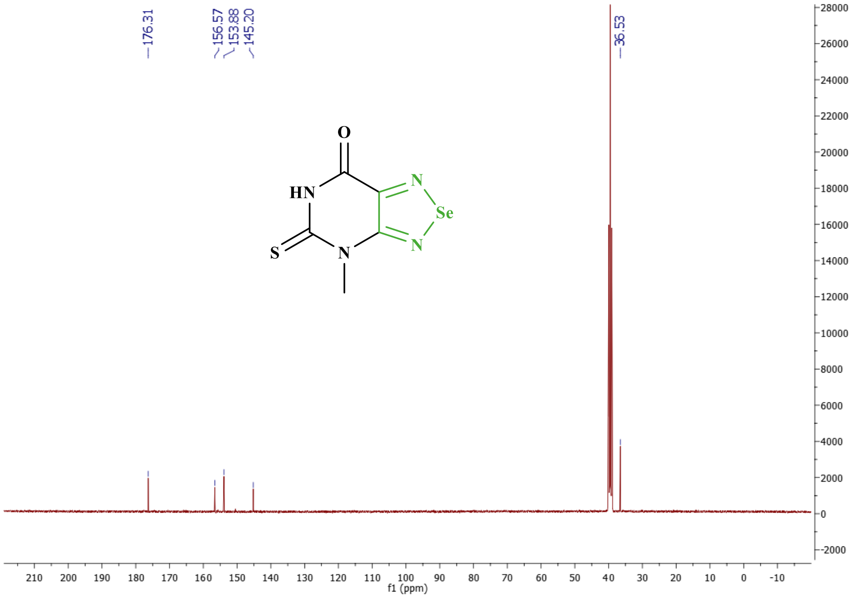

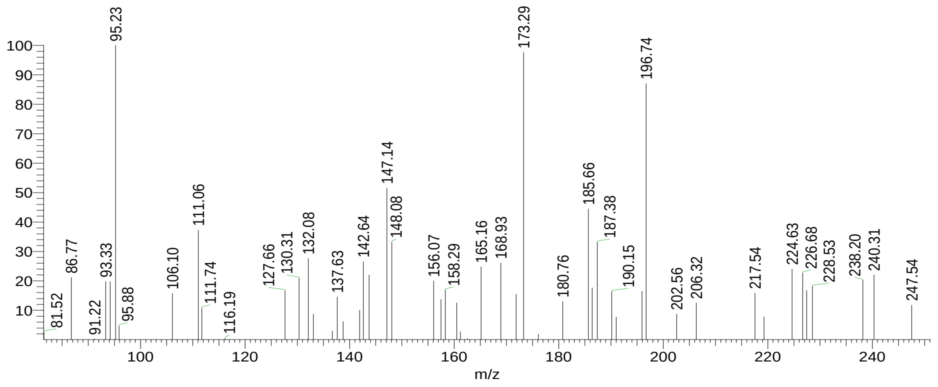

Figure S10: ^{1}H NMR, ^{13}C NMR, Mass and EDX spectra of 4-ethyl-[1,2,5]selenadiazolo[3,4-*d*]pyrimidine-5,7(4*H*,6*H*)-dione **(5c).**

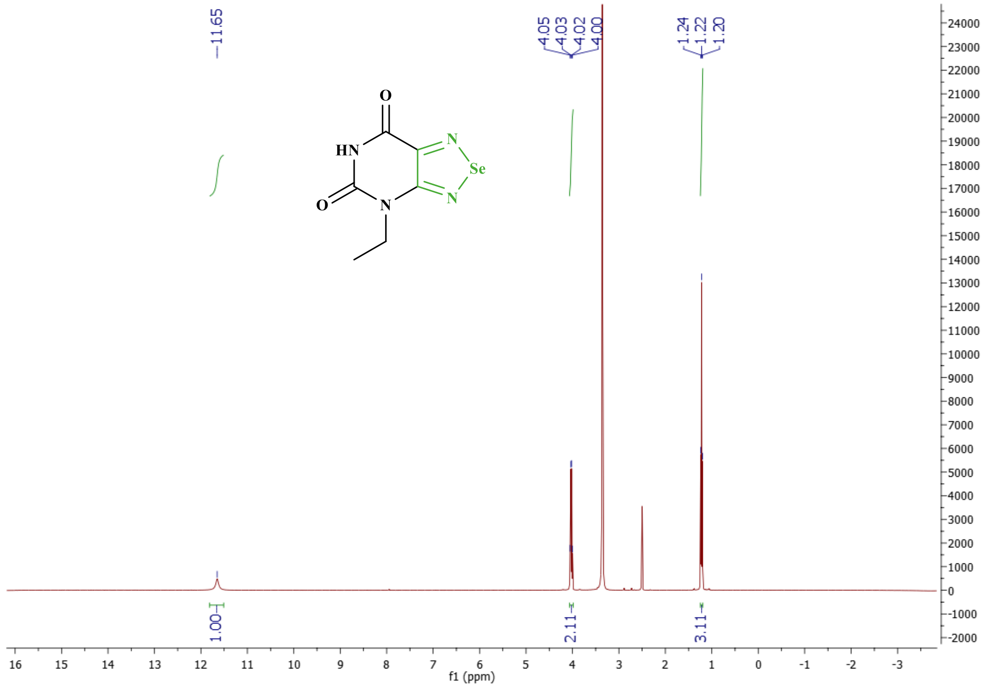

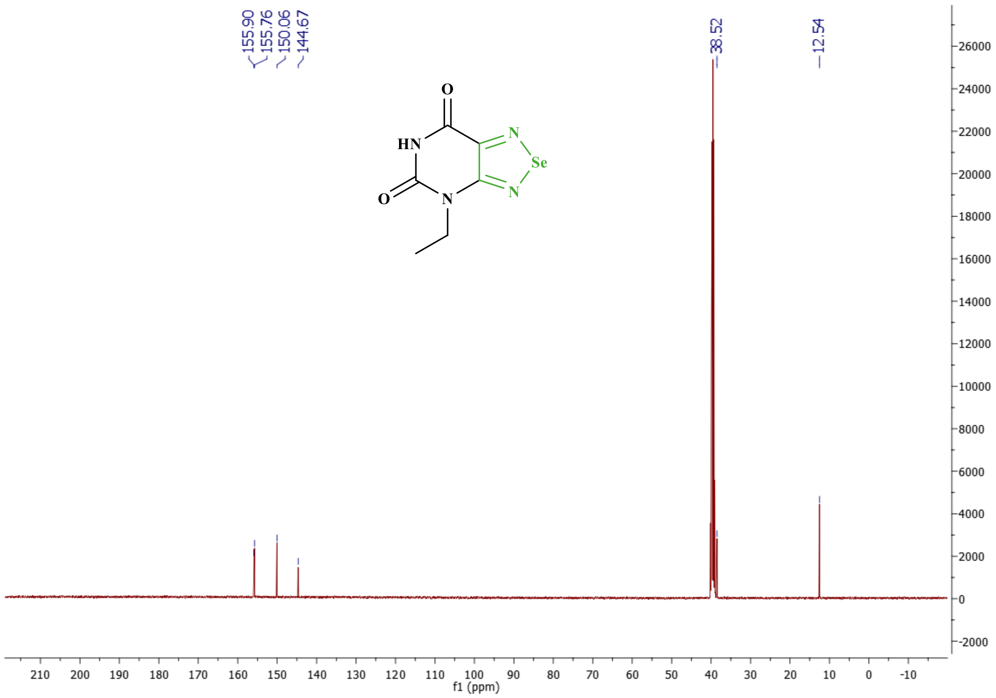

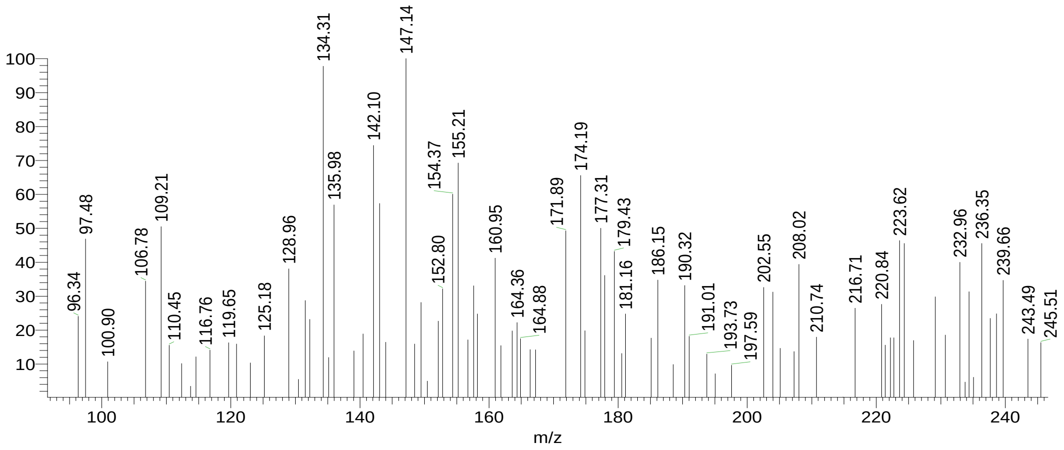

Application Note

5c

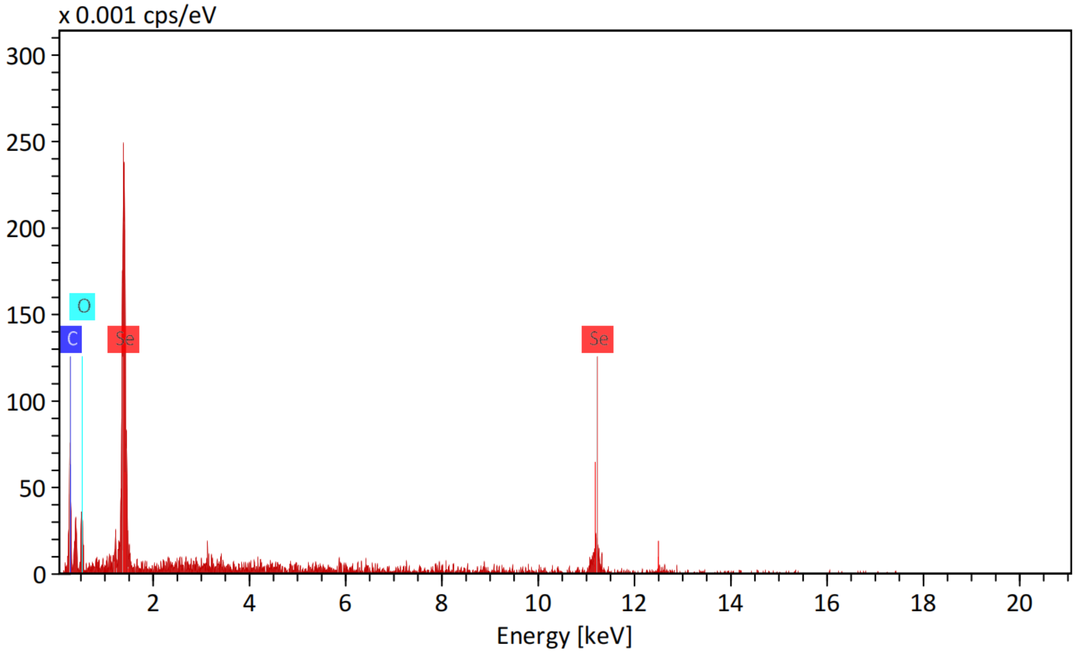

Figure S11: ^{1}H NMR, ^{13}C NMR and Mass spectra of 4-benzyl-[1,2,5]selenadiazolo[3,4-*d*]pyrimidine-5,7(4*H*,6*H*)-dione **(5d).**

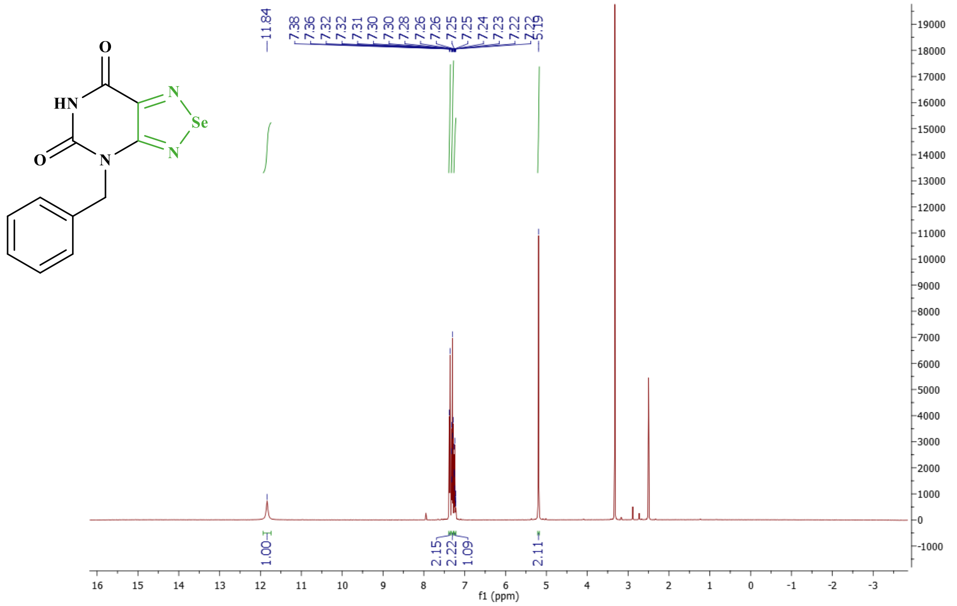

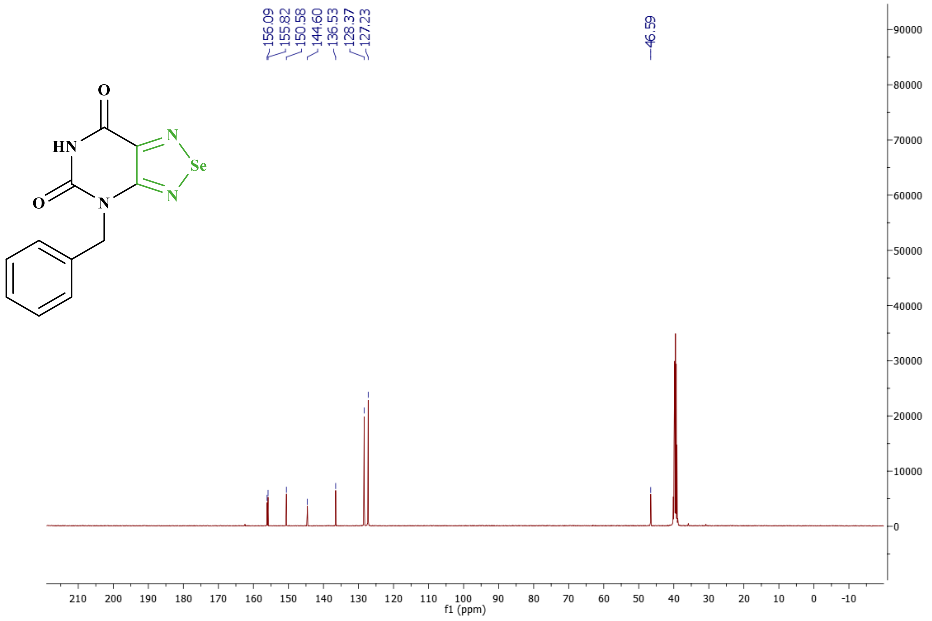

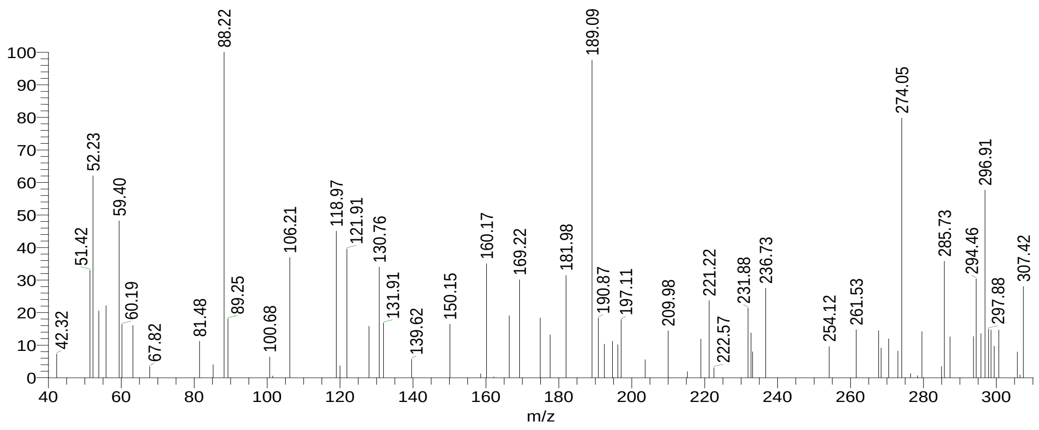

Figure S12: ^{1}H NMR, ^{13}C NMR, Mass and EDX spectra of 4-methyl-[1,2,5]selenadiazolo[3,4-*d*]pyrimidine-5,7(4*H*,6*H*)-dione **(5e).**

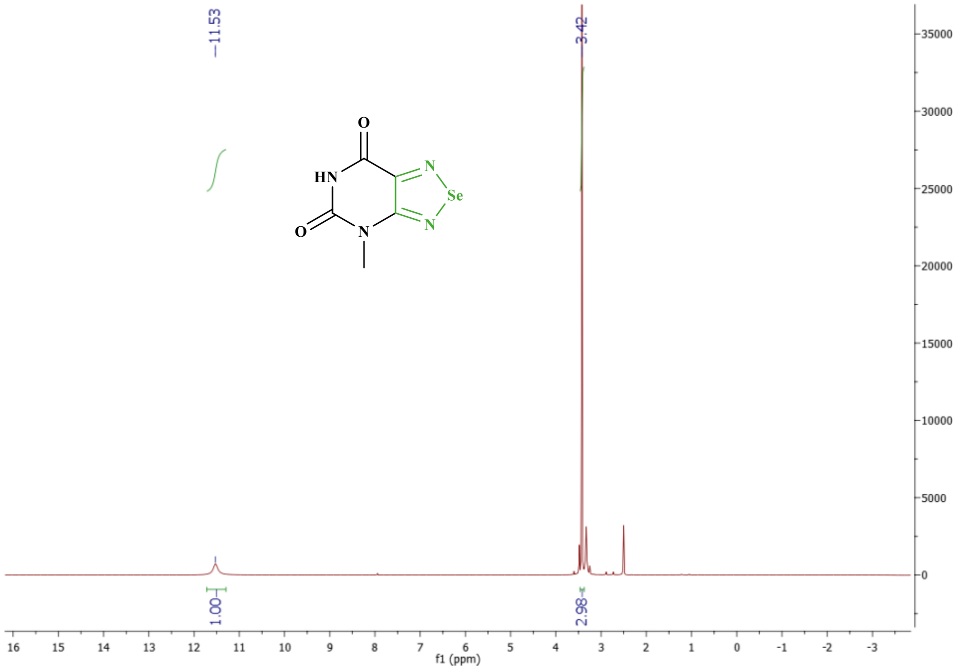

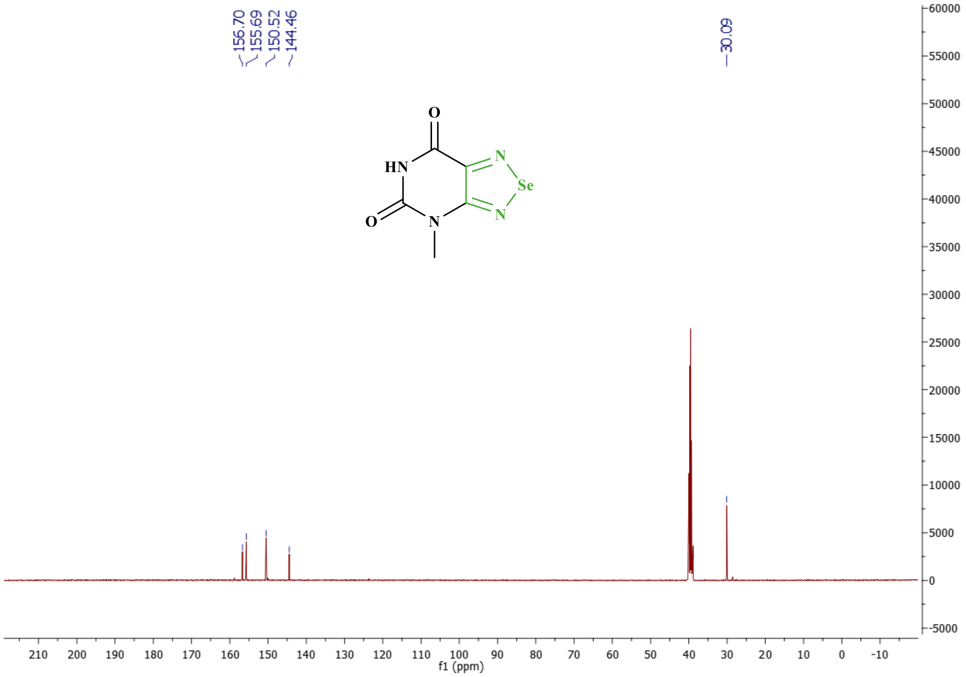

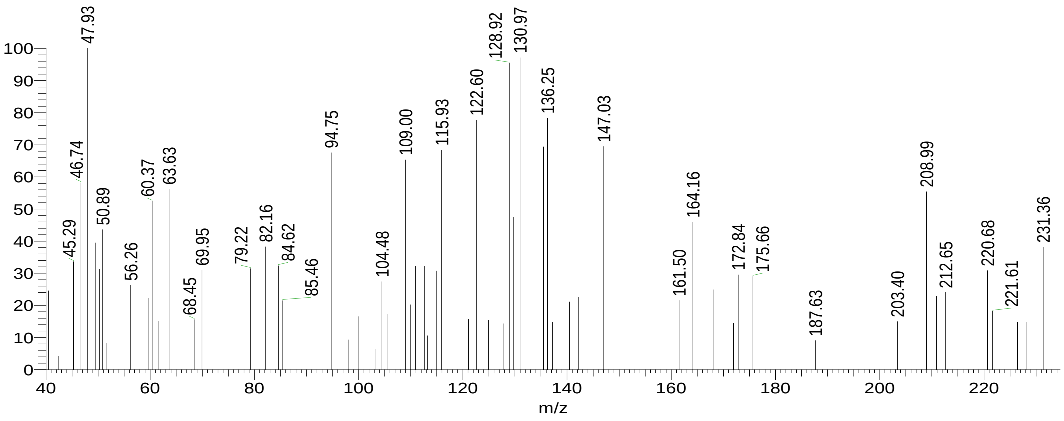

Application Note

5e

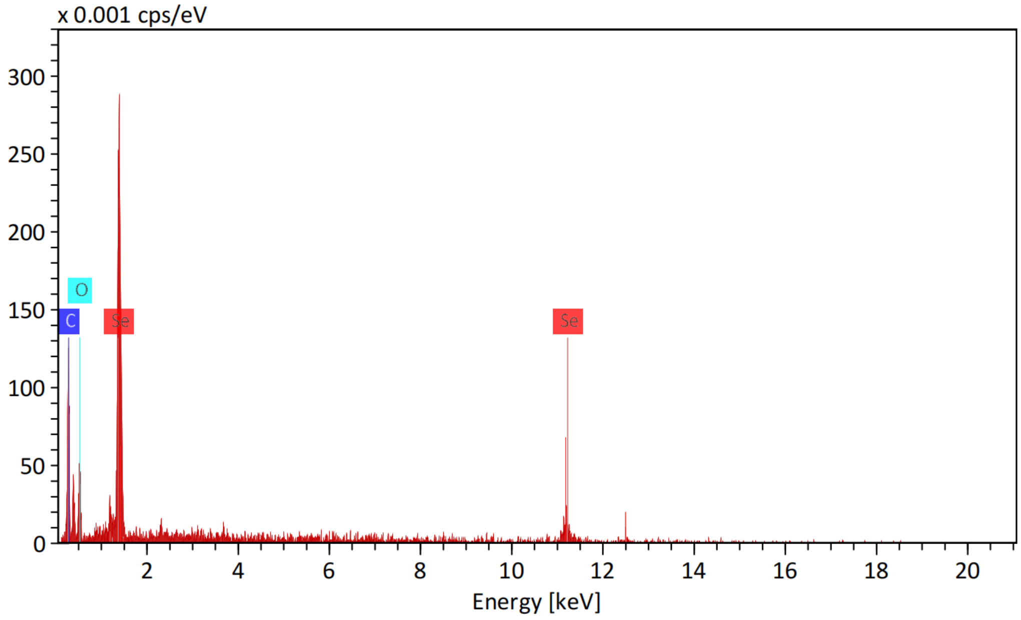

Figure S13: ^{1}H NMR, ^{13}C NMR and Mass spectra of 4,6-dimethyl-[1,2,5]selenadiazolo[3,4-*d*]pyrimidine-5,7(4*H*,6*H*)-dione **(7)**.

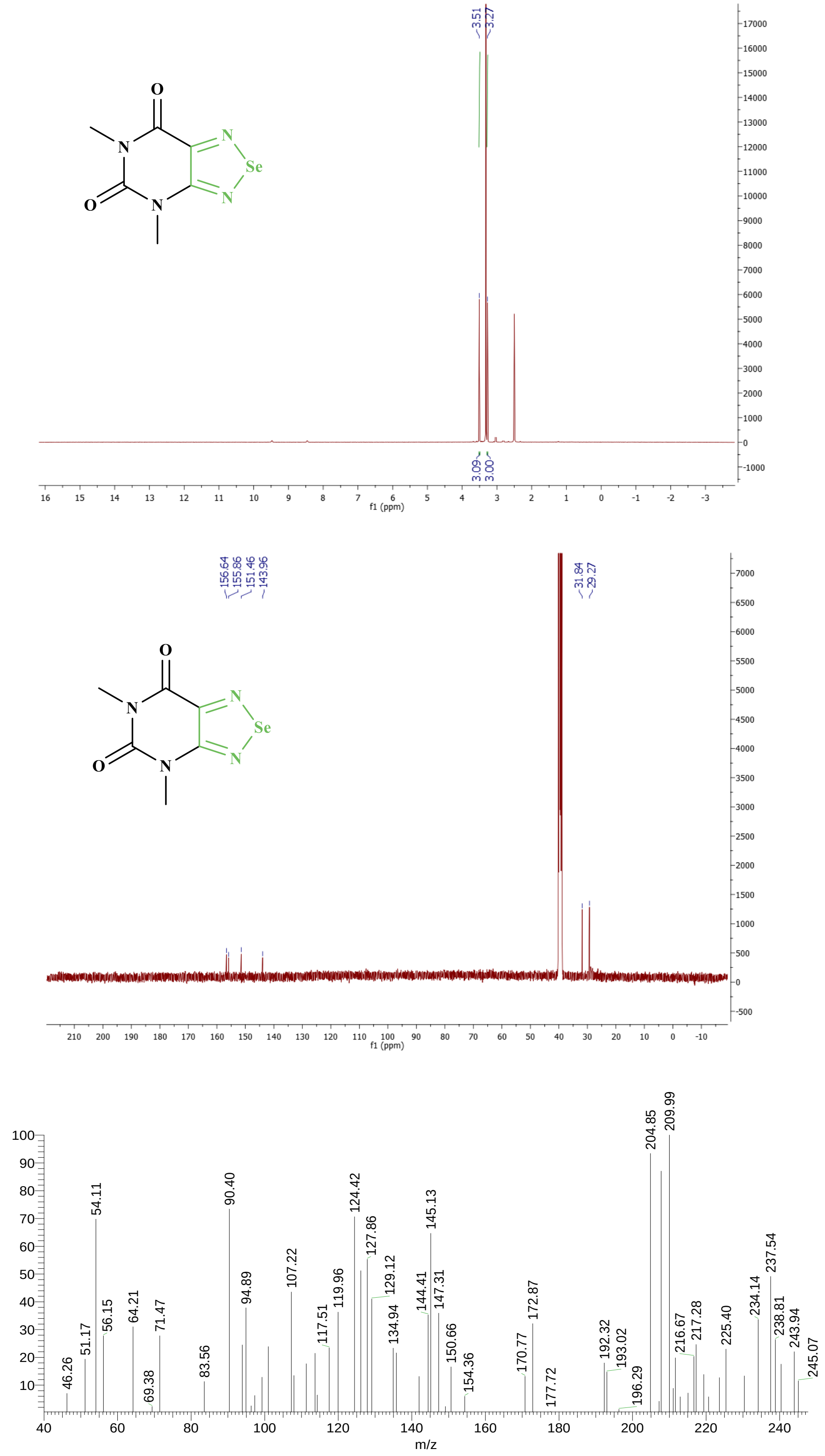

In silico physicochemical properties, drug-likeness data, and ADME profile for the all synthesized compounds compared to 5-Flourouracil

Table S1: Physicochemical properties and drug-likeness for the all synthesized compounds

Cpd. No.	M.W (g/mol)	ilogP	TPSA (Å)	HBA	HBD	RB	Lipinski violations
**3a**	354.40	2.09	109.98	3	3	4	0
**3b**	388.85	2.45	109.98	3	3	4	0
**3c**	294.37	1.96	125.00	2	3	2	0
**3d**	292.33	1.57	109.98	3	3	3	0
**3e**	264.28	0.80	120.84	3	4	2	0
**3f**	278.31	1.35	109.98	3	3	2	0
**5a**	215.07	-2.13	65.72	2	2	0	0
**5b**	245.16	-0.75	69.88	1	1	0	0
**5c**	243.12	-1.64	54.86	2	1	1	0
**5d**	305.19	-0.61	54.86	2	1	2	0
**5e**	229.09	-2.12	54.86	2	1	0	0
**7**	243.12	-2.11	44	2	0	0	0
**5-FU**	130.08	-0.73	65.72	3	2	0	0

Table S2: The ADME study results for the all synthesized compounds

Cpd. No.	solubility	BBB permeant	GI absorption	Cytochrome P450 (CYP inhibitor)			
				CYP2D6 inhibitor	CYP2C9 inhibitor	CYP2C19 inhibitor	CYP1A2 inhibitor
**3a**	-1.91	No	High	No			
**3b**	-4.07	No	High	No			
**3c**	-2.81	No	High	No			
**3d**	-2.26	No	High	No			
**3e**	-1.91	No	High	No			
**3f**	-1.87	No	High	No			
**5a**	-0.45	No	Low	No			
**5b**	-1.35	No	High	No			
**5c**	-0.80	No	High	No			
**5d**	-2.05	No	High	No			
**5e**	-0.41	No	Low	No			
**7**	-0.37	No	Low	No			
**5-FU**	-0.01	No	High	No			

Table S3: ***In-silico*** **toxicity properties for the all synthesized compounds**

Cpd. No.	Carcinogenicity	Ames test	Rat oral toxicity	Skin toxicity	HERG Blocker	Respiratory toxicity
**3a**	-ve	-ve	-ve	-ve	-ve	-ve
**3b**	-ve	-ve	-ve	-ve	-ve	-ve
**3c**	+ve	+ve	+ve	+ve	-ve	+ve
**3d**	-ve	-ve	-ve	+ve	-ve	+ve
**3e**	-ve	-ve	-ve	-ve	-ve	-ve
**3f**	-ve	-ve	-ve	-ve	-ve	-ve
**5a**	+ve	-ve	-ve	-ve	-ve	-ve
**5b**	+ve	+ve	+ve	-ve	-ve	+ve
**5c**	+ve	-ve	-ve	-ve	-ve	+ve
**5d**	+ve	-ve	-ve	-ve	-ve	-ve
**5e**	-ve	-ve	-ve	-ve	-ve	-ve
7	-ve	-ve	-ve	-ve	-ve	-ve
**5-FU**	-ve	-ve	+ve	-ve	-ve	-ve

Similar to 5-fluorouracil, all compounds showed no violation of Lipinski`s rule, where the molecular weight of compounds **3a-f**, **5a-e,** and **7** ranged from 215.07 g/mol to 388.85 g/mol. Compounds showed acceptable partition coefficients and topological polar surface areas ranging from -0.7 to 2.45 and 44 to 125, respectively, where log p and TPSA of 5-fluorouracil were -0.73 and 65.72, respectively. As for hydrogen bond donors and acceptable and rotatable bonds, compounds showed 0 to 4 rotatable bonds, 1 to 3 hydrogen bond acceptors, and 0 to 4 hydrogen bond donors following Lipinski`s rule of five (**Table S1**).

As for solubility, compounds were all soluble to very soluble with logS -4.0 to -0.37, similar to 5-fluorouracil, which had logS of -0.01 with very high solubility. Except for **5a**, **5e,** and **7**, all compounds showed high gastrointestinal absorption as designated by the white-boiled egg model, indicating the ability of compounds to be easily absorbed through the gut wall. Additionally, all compounds showed no ability to penetrate the blood-brain barrier and hence no CNS side effects as indicated by the yolk of boiled egg model similar to 5-fluorouracil. Also, compounds were suggested to show no side of potential liver toxicity as indicated by their non-inhibitory abilities of Cytochrome P450 (CYP2D6, Cyp2C9, Cyp2C19, and Cyp1A2) (**Table S2**).

The toxicity and carcinogenicity of compounds were studied using Admet lab0.2 online source. **3a** showed improved properties, including no possible carcinogenicity or mutagenicity as indicated by the Ames test, no acute oral toxicity, no respiratory, no skin toxicity, and no ability to block HERG enzyme that indicated no potential cardiovascular side effects. On the other hand, 5-fluorouracil showed potential acute oral toxicity in the rat model (**Table S3**).
